# What links ventilator driving pressure with survival in the acute respiratory distress syndrome? A computational study

**DOI:** 10.1186/s12931-019-0990-5

**Published:** 2019-02-11

**Authors:** Anup Das, Luigi Camporota, Jonathan G. Hardman, Declan G. Bates

**Affiliations:** 1 School of Engineering, University of Warwick, Coventry, UK; 2 Intensive Care Medicine, Guy’s and St Thomas’ NHS Foundation Trust and Division of Asthma Allergy and Lung Biology, King’s College London, London, UK; 3 Queen’s Medical Centre, Nottingham University Hospitals NHS Trust and School of Medicine, University of Nottingham, Nottingham, UK

**Keywords:** Mechanical ventilation, Driving pressure, Dynamic strain, Mechanical power, Tidal recruitment, Acute respiratory distress syndrome

## Abstract

**Background:**

Recent analyses of patient data in acute respiratory distress syndrome (ARDS) showed that a lower ventilator driving pressure was associated with reduced relative risk of mortality. These findings await full validation in prospective clinical trials.

**Methods:**

To investigate the association between driving pressures and ventilator induced lung injury (VILI), we calibrated a high fidelity computational simulator of cardiopulmonary pathophysiology against a clinical dataset, capturing the responses to changes in mechanical ventilation of 25 adult ARDS patients. Each of these in silico patients was subjected to the same range of values of driving pressure and positive end expiratory pressure (PEEP) used in the previous analyses of clinical trial data. The resulting effects on several physiological variables and proposed indices of VILI were computed and compared with data relating ventilator settings with relative risk of death.

**Results:**

Three VILI indices: dynamic strain, mechanical power and tidal recruitment, showed a strong correlation with the reported relative risk of death across all ranges of driving pressures and PEEP. Other variables, such as alveolar pressure, oxygen delivery and lung compliance, correlated poorly with the data on relative risk of death.

**Conclusions:**

Our results suggest a credible mechanistic explanation for the proposed association between driving pressure and relative risk of death. While dynamic strain and tidal recruitment are difficult to measure routinely in patients, the easily computed VILI indicator known as mechanical power also showed a strong correlation with mortality risk, highlighting its potential usefulness in designing more protective ventilation strategies for this patient group.

**Electronic supplementary material:**

The online version of this article (10.1186/s12931-019-0990-5) contains supplementary material, which is available to authorized users.

## Background

Acute Respiratory Distress Syndrome (ARDS) is characterized by acute heterogeneous inflammation of the lungs leading to deterioration in effective gas exchange [1], worsening of lung compliance [2], increasing pulmonary shunt [3], and non-cardiogenic pulmonary oedema caused by increased pulmonary vascular permeability [4]. Almost 1 in 10 intensive care admissions meet the ARDS criteria, 70% of which are categorized as ‘moderate’ or ‘severe’ ARDS, with mortality rates exceeding 40% [5]. The most common means of reinstating adequate oxygenation in patients with ARDS is through mechanical ventilation (MV), with the aim of meeting patients’ oxygenation and CO_2_ removal requirements while the underlying pathology is treated. However, the lungs of patients suffering from ARDS are highly susceptible to damage introduced by mechanical ventilation, commonly known as *ventilator-induced lung injury* (VILI). Minimizing lung injury, while maintaining adequate gas exchange (“protective ventilation”), is thus a crucial requirement in the effective clinical management of ARDS. Currently, there is widespread confusion regarding how best to achieve protective ventilation in ARDS, with multiple debates ongoing regarding the problem. Many strategies have been proposed, and are in use; each with its own rationale, advocates and evidence of effectiveness [6–11]. Evaluating the relative benefits of different strategies using only traditional approaches (e.g. randomized trials) is challenging and costly, due to the number of potential strategies and disease states, and to the difficulty of recruiting critically ill patients to clinical trials.

Recently, some strong evidence associating airway driving pressure with patient mortality in ARDS was provided in two different secondary analyses of previously performed clinical trials [12, 13]. Airway-driving pressure (ΔP) in controlled mechanical ventilation is defined as the difference between plateau pressure and total PEEP or as the ratio of tidal volume (V_T_) divided by respiratory-system compliance (C_RS_). Using a statistical tool known as multilevel mediation analysis, Amato and colleagues analyzed individual data from 3562 patients with ARDS enrolled in nine previously reported randomized trials, and found that a change in ΔP post-randomization was the ventilator variable (mediator) that best stratified relative risk of death. A reduction in ΔP owing to changes in ventilator settings was strongly associated with increased survival – independently from the randomization process [12]. Although causality could not be established, the authors speculated that ΔP could be a surrogate for dynamic lung strain, and that dynamic strain might predict mortality-associated lung injury better than other physiological variables. It is also possible that – as ΔP is mathematically coupled with tidal volume and elastance – the value of ΔP only reflects a change in lung mechanics (i.e., elastance) which follows a change in ventilator settings, and therefore setting a specific value of ΔP may not per se decrease the risk of death [13, 14].

Here, we investigate the physiological mechanisms underlying the above questions using a comprehensive and well-established computer simulation of integrated cardiopulmonary pathophysiology in individual ARDS patients. Twenty-five different in silico patients were simulated by independently calibrating the simulator to a clinical dataset of twenty-five adult ARDS patients, capturing the key responses to changes in mechanical ventilation. Each of these in silico patients was then subjected to the same range of values of driving pressure and positive end expiratory pressure (PEEP) suggested in [12]. The key advantage of this approach is that, in contrast to statistical analyses and clinical trials, it allows us to “look inside” the lung, and investigate the precise relationship between ventilator settings, key physiological variables, and indices of VILI. By comparing the changes in these variables over different values of ΔP and PEEP with the corresponding data on patient mortality published in [12], we can investigate possible physiological mechanisms that could link driving pressure with relative risk of mortality in ARDS, and hence evaluate which VILI indicators correlate best with patient outcomes in this context.

## Methods

### Computational model of cardio-pulmonary physiology

The study employs a multi-compartmental computational model that simulates integrated pulmonary and cardiovascular pathophysiology [15–17]. The core models have been designed to represent a dynamic in vivo cardiovascular-pulmonary state, comprising conducting airways and a respiratory zone of 100 parallel alveolar compartments, with each compartment having a corresponding set of parameters accounting for stiffness, threshold opening pressures (TOP) and extrinsic pressures as well as airway and peri-alveolar vascular resistances. This allows for a wide spectrum of ventilation perfusion mismatch to be replicated. The model includes inherent physiological reflex mechanisms, e.g. hypoxic pulmonary vasoconstriction. The mathematical and physiological principles on which the simulator is based have been detailed in previous studies [18–21], which have also validated the simulator’s capability to represent the pulmonary disease states of individual patients with chronic obstructive pulmonary disease and ARDS [15, 22]. A detailed description of the mathematical equations implementing the physiological aspects of the computational model is provided in the Supplementary Material.

### Patient data and simulation

Data were extracted for adult 25 ARDS patients from Borges et al. [23]**.** The PF ratio (ratio of partial pressure of arterial oxygen to fraction of oxygen in inspired air) was used to assess the severity of ARDS in the patients, in accordance with the Berlin definition [24]. Of the total number of simulated patients, 13 were classified as severe (PF ratio < 100 mmHg), 7 were classified as moderate (PF ratio 100–200 mmHg) and 6 were classified as mild (PF ratio > 200 mmHg). An ideal body weight of 70 kg was assumed for all patients. All patients were assumed to be fully sedated and requiring full respiratory support through positive pressure mechanical ventilation.

The two main models of the simulator, the pulmonary model and the cardio-vascular model, were calibrated against the dataset from Borges et al. [23], using a genetic-algorithm based optimization strategy, as previously described in [17]. The key pulmonary model parameters assign airway resistances, compliances, and threshold opening pressures to each alveolar unit and also determine the characteristics of the conducting zones. These were identified at 10 cmH_2_O of PEEP, where model outputs of PF ratio, arterial carbon dioxide tension, mixed venous oxygen saturation (SvO_2_), and static compliance (Cstat) were matched to baseline data given in Borges et al. [23]. Next, the parameters of the cardiovascular (and the integrated cardio-pulmonary model), such as those governing the properties of cardiac chambers, major arteries and major vessels, were identified by matching the model outputs for cardiac index (CI), arterial carbon dioxide tension (PaCO_2_), mean arterial pressure (MAP) and mixed venous oxygen saturation (SvO_2_) to data for varying PEEP levels, during the administration of a recruitment maneuver. All model parameters were constrained to vary between appropriate physiological ranges. Full details of the model calibration process are provided in the Supplementary Material.

### Measurements of key physiological variables and VILI indices

After establishing the capability of the simulator to reproduce the responses of all 25 ARDS patients to variations in ventilator settings, measurements were made of changes in all relevant physiological variables as the in silico patients were subjected to the same levels of driving pressure and PEEP that were analyzed in the study of Amato and colleagues [12]. Driving pressure was determined as the difference between the plateau pressure and the PEEP. To observe the cardio-pulmonary effects of interest, the following values were recorded: arterial oxygen tension (PaO_2_), arterial carbon dioxide tension (PaCO_2_), arterial pH (pH), arterial oxygen saturation (SaO_2_) and SvO_2_, volume of individual alveolar compartments at end of inspiration and end of expiration (V_alv_insp_ and V_alv_exp_, respectively), and pressure of individual compartments at end of inspiration and end of expiration (P_alv_insp_ and P_alv_exp_, respectively), cardiac output (CO; cardiac index was calculated from CO indexed to body surface area), the mean arterial pressure (MAP), the mean pulmonary artery pressure (MPAP) and oxygen delivery (DO_2_; using the values of CO, SaO_2_ and PaO_2_ and given hemoglobin level (Hb)). Recruitment was calculated as the fraction of alveoli receiving zero ventilation subsequently achieving ventilation. Several proposed indices of VILI were calculated, e.g. respiratory system compliance (*C*_*RS*_) [25], intra-tidal recruitment [26, 27], mechanical power [28], mean alveolar pressure (*P*_*alv*_) [29], and the strain on the lung (both dynamic and static [30]). *C*_*RS*_ is calculated as ∆V/ (P_plat_ - PEEP), where P_plat_ is the plateau pressure. The dynamic strain is calculated as ∆V / V_frc_, where V_frc_ is V_alv_exp_ at PEEP = 0 cmH_2_O and ∆V = V_alv_insp_-V_alv_exp_. Static strain was calculated as V_alv_exp_/ V_frc_. Intra-tidal recruitment was calculated as the difference between the fraction of total ventilated lung at the end of inhaling and end of exhaling, expressed as a percentage. The mean alveolar pressure, *P*_*alv*_, is calculated as the average end-inspiratory pressure (of the highest 20% values) distributed across the lung, which is a better indicator for end-inspiratory lung pressure than the often used surrogate, plateau pressure. Gattinoni and colleagues [28, 31, 32] have suggested that instead of separately considering the mechanical factors (pressure, volume, rate and flow) associated with VILI, it may be better to combine these factors as the ‘mechanical power’ imparted to the lung by the ventilator, which we calculated using the following equation (Eq. 6 from [28]):

$$ \underset{J\ {\mathit{\min}}^{-1}}{\mathrm{Power}}=0.098. VR.\left\{{V_T}^2.\left[\frac{1}{2}.E+ VR.\frac{\left(1+ IE\right)}{60. IE}.{R}_{aw}\right]+{V}_T. PEEP\right\} $$

Here, *VR* is the respiratory rate set by the ventilator, *V*_*T*_is the ventilator delivered tidal volume, *E* is the elastance of the respiratory system (calculated as 1/*C*_*RS*_), *IE* is calculated as the inhalation time/ exhalation time in a single breath, *R*_*aw*_ is the airway resistance calculated using the difference in peak pressure and plateau pressure at the ventilator and resultant flow (*V*_*T*_ / inspiratory time) during a breath, and *PEEP* is the total positive end-expiratory pressure. During simulations, all parameters were recorded every 10 milliseconds. All simulations, model calibration and data analysis were performed within the Matlab® 2015a programming environment.

## Results

### Computational model calibration results

**Fig. 1:**
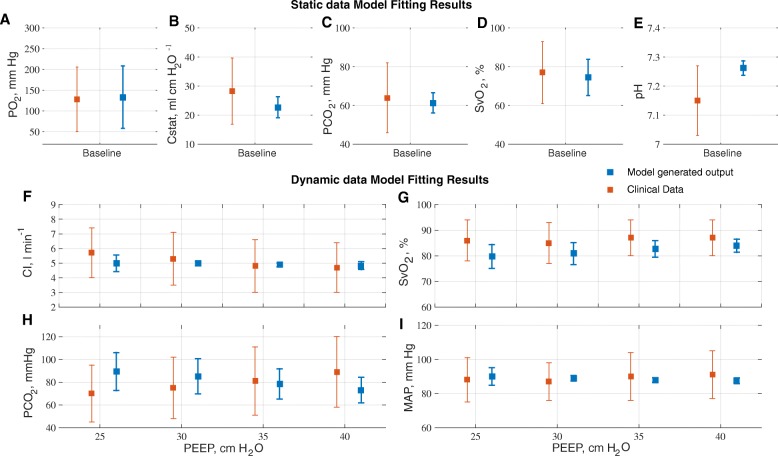
Results of fitting patient data to model to acquire virtual ARDS subjects. Subplots **a-e** show the results of fitting the model to static data and **f-i** show the results of fitting the model to dynamic data. All error bars show the mean and 1 standard deviation of clinical data (Borges 2006) in red and corresponding model outputs in blue. PO_2_ - partial pressure of oxygen in arterial blood, Cstat - static Compliance, PCO_2_ - partial pressure of carbon dioxide in arterial blood (mm Hg), SvO_2_ - oxygen Saturation in mixed venous blood, pH - pH of arterial blood, CI - cardiac Index (ml min^− 1^ m^− 2^), MAP - mean arterial pressure (mm Hg)

The results of calibrating the model against data on 25 ARDS patients reported in Borges et al. [23] are given in Fig. 1. All model outputs of interest were consistently very close to the values reported in the clinical data (overall r^2^ > 0.94, *p* < 0.00001), thus supporting the ability of the simulator to reproduce the physiological responses of each individual patient. Figure 1 reports the mean and standard deviation for the data and model outputs across the patient cohort. A comparison of model outputs versus individual patient data for all 25 patients is provided in the Supplementary Material. Baseline characteristics of the simulated patients are listed in Table 1.

**Table 1:** Characteristics of in silico patients at baseline

	Baseline
	Mean (SD)
PaCO_2_, mmHg	61 (5.2)
pH	7.24 (0.03)
SvO_2_, %	74 (9.3)
Shunt Fraction, %	34 (11.3)
CI, L min^− 1^ m^− 2^	5 (0.3)
MAP, mm Hg	90 (2.6)
PF ratio, mmHg	133 (75)
- Mild	244 (37)
- Moderate	152 (37)
- Severe	72 (9.6)
Cstat, ml cmH_2_O^−1^	22 (3.6)
*VR*, breaths min^−1^	12.5 (0.8)
*V*_*T*_, ml PBW	5.9 (0.08)

CI - Cardiac Index (indexed to body surface area), FiO_2_ - Fraction of O_2_ in inspired gas, PEEP - Positive End Expiratory Pressure, PF ratio – Ratio of Arterial oxygen tension to FiO_2_, PaCO_2_ - Arterial carbon dioxide tension, SvO_2_ - Mixed Venous Oxygen Saturation, Cstat – Static Compliance, MAP – Mean Arterial pressure, *VR* – Respiratory Rate, *∆V* – Tidal Volume

Baseline in silico patients at PEEP 5 cm H_2_O, FiO_2_ 1.0, plateau pressure 30 cmH_2_O. PF ratio and Cstat recorded at PEEP 10 cmH_2_O (as given in (*15*))

### Comparing VILI indices with mortality risk for different ΔP and PEEP settings

**Fig. 2:**
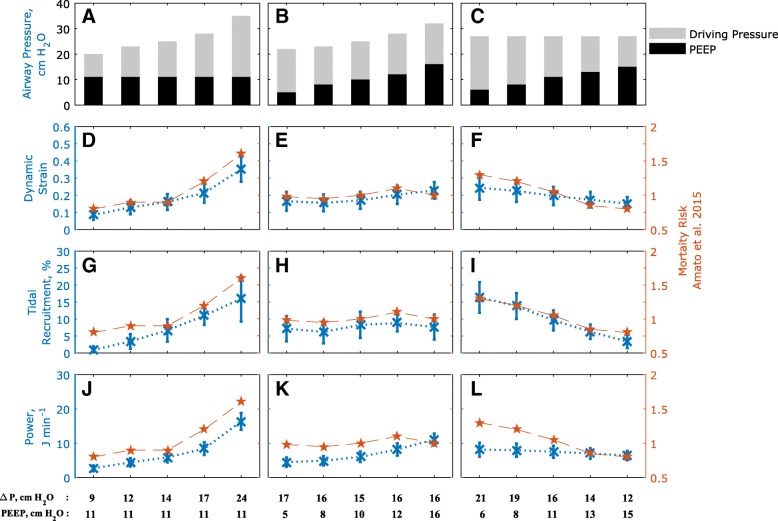
Results of changing driving pressure and PEEP (**a-c**) on dynamic strain (**d-f**), intra-tidal recruitment (**g-i**) and mechanical power (**j-l**). Subplots **d**–**i** also indicate the mortality risk rates (red) published in Amato et al. (12) for the same changes in airway pressure and PEEP (**a-c** and listed at the foot of the figure) shown here. Blue crosses show mean values in the population and the error bars represent 1 standard deviation

**Fig. 3:**
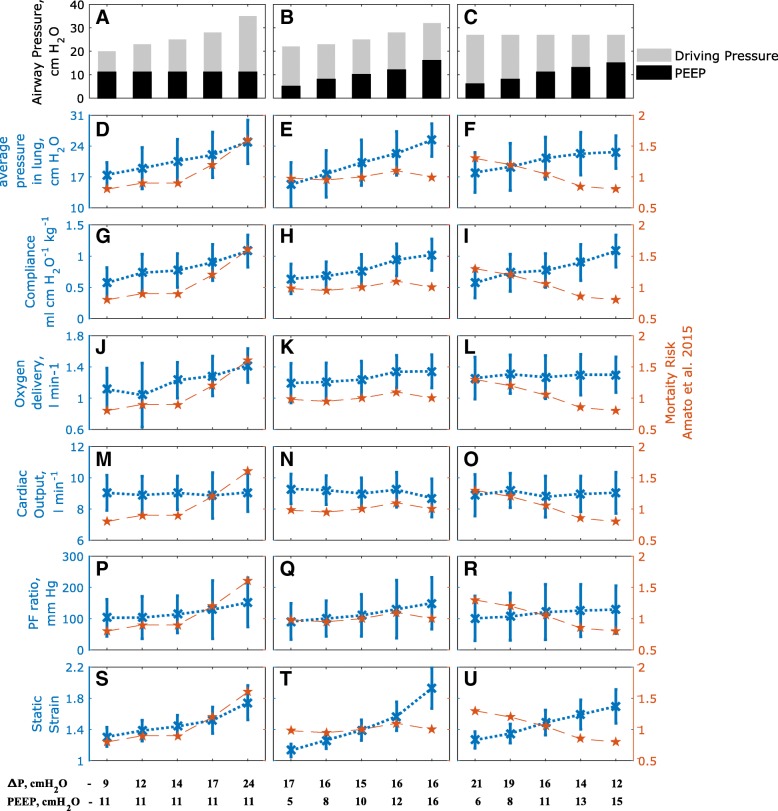
Results of changing driving pressure and PEEP (**a-c**) on alveolar pressure (**d-f**), dynamic compliance (**g-i**), peripheral oxygen delivery (**j-l**), cardiac output (**m-o**), PF ratio (**p-r**) and static strain (**s-u**). Subplots (**d-u**) also indicate the mortality risk rates (red) published in Amato et al. [12] for corresponding changes in airway pressure (subplots **a–c** and listed at the foot of the figure). Blue crosses show mean values in the population and the error bars represent 1 standard deviation

Figures 2 and 3 show the responses of key physiological variables (in blue) for the same variations in ΔP and PEEP levels used in [12], across the patient cohort. Figures 2 and 3 also show the relative risk of mortality (in red) as reported in [12], for the corresponding ΔP /PEEP levels, with values higher than 1 indicating increased mortality rate after multivariate adjustment (for details see supplementary material of [12]). Of all indices of VILI investigated, only dynamic lung strain, mechanical power, and tidal recruitment were strongly positively correlated (r > 0.85, *p* < 0.05) with the mortality risk to changes in ΔP reported in [12] (Table 2). Larger ΔP values, while keeping PEEP constant, led to significant increases in dynamic strain (Fig. 2d), mechanical power (Fig. 2j) and tidal recruitment (Fig. 2g) that were strongly and significantly correlated with increases in relative risk of mortality, (r = 0.99, 0.99 and 0.96 respectively, with p < 0.05). On the other hand, higher plateau pressures caused by using higher PEEP values while keeping ΔP constant, resulted in very small increases in dynamic strain and tidal recruitment, consistent with the lack of effect on mortality risk reported in [12] (Fig. 2e, h, k). Finally, higher PEEP values combined with lower ΔP led to significant decreases in dynamic strain (Fig. 2f), mechanical power (Fig. 2l) and tidal recruitment (Fig. 2i), which were strongly correlated with the decreased relative risk of mortality reported in [12] (r > 0.95, *p* < 0.01 for all cases).

**Table 2:**
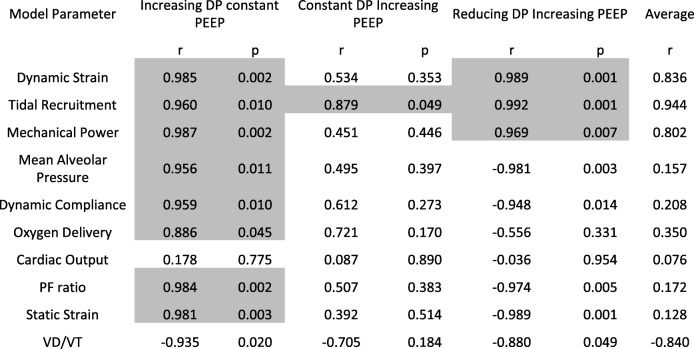
Correlation coefficients of outputs with respect to mortality data

The table reports the Pearson linear correlation coefficient (r) between the simulated outputs and mortality data reported in 12, with corresponding p value using the students t distribution. Shaded areas highlight parameters with strong positive and significant correlations with mortality data (r > 0.85, *p* < 0.05). Table ordered in descending order for average r. PF ratio is the ratio of arterial oxygen tension to fraction of O_2_ in inspired air. VD/VT is the physiological deadspace fraction

Other potential VILI indices, including dynamic compliance, alveolar pressure and static strain, were also calculated, but showed significant deviations from the data on patient mortality as driving pressure and/or PEEP were varied. They are illustrated in Fig. 3 along with peripheral oxygen delivery, cardiac output and PaO_2_/FiO_2_ (PF) ratio, with corresponding correlation coefficients reported in Table 2. Increases in lung compliance, average pressure across the lung, static strain and PF ratio were observed with increasing PEEP or driving pressure. Lowering driving pressures by increasing PEEP did not result in any adverse outcomes with respect to oxygenation (Fig. 3l, r). There were no significant effects on cardiac output in all three interventions (Fig. 3m-o). Static strain rose considerably across the three interventions (Fig. 3s-u), but the biggest rise was observed in the constant ΔP with higher PEEP group (Fig. 3t). Individual results for all 25 patients are reported in the supplementary material.

## Discussion

The current standard recommendation for providing lung protective ventilation in patients with ARDS supports the use of low tidal volumes (LTV) while integrating higher levels of PEEP. In the seminal ARDS Network (ARDSnet) study [33], this LTV protective strategy resulted in significantly improved survival rates when compared to the then ‘traditionally-used’ higher tidal volumes. Since then, however, various studies have failed to show further uniform benefits of LTV in ARDS patients [10, 11, 34]. These studies found that even with the use of the proposed ARDSnet protocol, the mortality rate in ARDS patients remained relatively high (> 40% [34]), which was attributed to the probable tidal hyperinflation of the normally aerated part of the lungs. By itself, therefore, it seems clear that LTV is not sufficient to achieve a lung protective ventilation. In 2015, Amato and colleagues [12] presented the results of a retrospective analysis of individual patient data, with the conclusion that the driving pressure (tidal volume normalized to lung compliance) was better associated to survival in ARDS patients than either tidal volume or plateau pressure separately. Following this, various studies have corroborated those results [13, 35], and shown that driving pressure was a good indicator of lung stress [36], higher driving pressures were associated with more postoperative pulmonary complications [35], and higher driving pressures may induce lung injury more easily in patients with low respiratory system compliance [37]. The results in this paper support the hypothesis that higher driving pressure results in increases in several important indices of VILI.

More recently, a trial involving a patient cohort of 1010 patients with moderate to severe ARDS found that the use of higher PEEP values (titrated to optimal respiratory system compliance) without significant deviations in driving pressure, were associated with higher risk of mortality [38]. In our virtual patient cohort, this ventilation strategy was associated with significant increases in mechanical power (Fig. 2k), alveolar pressure (Fig. 3e) and static strain (Fig. 3t). The lack of significant change in overall mortality in the Amato study [12] in this case may be due to the significant number of mild ARDS patients in that cohort, who would have tolerated the higher plateau pressures better. At the same time, our results indicate that increases in PEEP that were accompanied by significant decreases in ΔP result in reductions in the intra-tidal opening and closing of lung units (Fig. 2l), together with increased lung compliance (Fig. 3i). These results lend further weight to the argument that the key goal in attempting to provide more protective ventilation should be to achieve significant reductions in driving pressure.

Many different potential physiological indicators of VILI have been proposed in the literature, but in almost all cases, their direct effect on patient mortality has not been conclusively established. Furthermore, ventilator effects leading to hemodynamic impairment are rarely considered in the context of VILI, even though pulmonary vascular dysfunction and acute cor pulmonale are well recognized outcomes in ARDS [39, 40], even during protective ventilation [41, 42]. By inserting a detailed model of cardiopulmonary physiology between the data linking ventilator settings and relative risk of mortality published in [12], we can investigate which physiological mechanisms correlate best with patient mortality. With respect to VILI, markers of lung injury attempt to quantify the potentially destructive contribution of mechanical forces to exacerbating pre-existing lung pathology. One such marker is the dynamic lung strain [43], which accounts for the increase in tissue tensions and detrimental effects of dynamic tidal excursions. The results of this paper suggest that dynamic strain correlates well with the changes in mortality rates observed for varying values of driving pressure. The authors of [12] recognize this as a possible explanation for their findings, and the results provided here provide further support for this hypothesis.

Although our results indicate that dynamic strain provides a plausible physiological mechanism to explain the effect of driving pressure on patient mortality, this is not a quantity that can be readily and accurately calculated by clinicians at the bedside. There is also a reasonable argument that it is the combination of multiple dynamic components of ventilation, e.g. frequency, strain rate, etc. alongside variables such as stress and strain that may accentuate the risk of lung injury in ARDS patients [44]. Gattinoni and colleagues [14, 31] have proposed the concept of ‘mechanical power’ as a determinant of VILI, combining key ventilator settings and lung mechanics into a single mathematical equation which can be readily computed. The results presented in Fig. 2 and Table 2 show mechanical power to be strongly correlated with mortality rates published in [12] for the given changes in driving pressures and PEEP.

It should be noted from Fig. 2 that the only ΔP /PEEP values for which the mechanical power was significantly higher than 12 J/min (Fig. 2i) (the maximum value recommended by Guerin et al. [13], who associated staying below this threshold with higher survival rates) was at a driving pressure of 24 cmH_2_O and PEEP of 11 cm H_2_O. It can be clearly seen across the cohort that despite the overall increase in PEEP and plateau pressures, the improvement in lung compliance (Fig. 3i) and lower driving pressures do not result in a significant rise in delivered mechanical power. It should be noted that the investigation in this paper was limited to comparing the effects of the driving pressure values given in [12], which did not account for mechanical power or explicitly report key components for calculating power, i.e. respiratory rate, duty cycle, airway resistances etc. The overall positive correlation to mortality rates (Table 2), however, supports mechanical power as a potentially important indicator of lung injury in ARDS patients.

Dynamic strain accounts for normally-aerated lung regions in ARDS [45] as well as the intra-tidal recruitment of alveolar units. However, studies [46] have suggested that strain and intra-tidal opening and closing of alveolar units should be considered independently. In clinical investigations, the amount of recruited lung is often determined using whole lung CT scans and chest x-ray, or estimated using pressure volume curves. The computational model used in our study allows for the direct and continual assessment of the total recruited lung region - the resulting measure of ‘tidal recruitment’ (the amount of newly recruited lung during tidal ventilation) is a distinctive indicator for VILI. The results of this study show a strong correlation between the reported mortality data and tidal recruitment for the investigated variations in driving pressure (r > 0.85, *p* < 0.05 in all strategies). The results indicate that reducing ΔP from 21 cm H_2_O to 12 cm H_2_O reduced intra-tidal opening and closing of alveolar units from an average of 16% to below 4% of the total lung. Tidal recruitment thus offers another potentially valuable marker for lung injury in ARDS for investigation in future prospective studies.

Several other important physiological variables were also evaluated in this study; including lung compliance, end inspiratory lung pressures, PF ratio, static strain, cardiac output and oxygen delivery to peripheral tissue. Generally, the correlation between these indicators and reported mortality rates observed with respect to changes in ΔP was weak. As expected, the average lung pressures increased uniformly across the three interventions accompanying increases in ventilator pressures. Lung compliance, PF ratio, and static strain also increased with ventilator pressures, indicating better-aerated regions, improved ventilation/perfusion mismatch and increased end-expiratory lung volumes, respectively.

The study described in this manuscript has some limitations. The patient data used was collected from patients with high baseline values of cardiac output (CO). These values are consistent with the data in [23], which reported a mean cardiac index of 5.8 l min^− 1^ m^− 2^ at plateau pressure of 30 cmH_2_O in their patient cohort. The ability to draw conclusions about the precise presence or absence of cardiopulmonary dysfunction is limited from the information available in the published data sets. However our simulations do exhibit the cyclical changes in venous, ventricular and arterial systems observed in response to positive pressure ventilation [16, 17, 47]. Parameters such as ventilation rate (VR) and duty cycle (DC) were left at baseline settings (determined at model calibration stage) throughout the simulations, due to limited availability data and no systematized guidelines available for prospective ΔP adjustment. It should also be noted that the studies that provided the data used for model calibration reported no significant changes in heart rate throughout their interventions, making it likely that the drugs and dosages used for sedation suppressed normal cardiovascular system baroreceptor reflexes. As such, autonomic reflex modules of the model could not be calibrated, and hence were not utilized. The measured alveolar strain was used as a surrogate for the effects of increased inflammatory mediators found in ARDS, whose effects are otherwise difficult to isolate and quantify in clinical settings [48]. Finally, any computational model will be an approximation of the relevant physiological processes and cannot reflect all the inherent complexity of the underling pathophysiology.

## Conclusions

It is clear that ARDS encompasses multiple different aetiologies, with different outcomes, and thus mortality rates can never be determined solely by any single factor, including VILI. Nevertheless, the results in this paper suggest a plausible physiological basis for the recently proposed link between driving pressure and mortality in ARDS, that could be tested in future clinical studies. Using a high-fidelity computational simulator of cardiopulmonary pathophysiology, we observe that cyclic alveolar strain combined with tidal recruitment may provide a credible mechanistic explanation for the proposed association between higher driving pressures and greater relative risk of death in ARDS. While these indices are difficult to measure directly in patients, the easily-computed VILI indicator known as mechanical power also showed a strong correlation with mortality risk, highlighting its potential usefulness in testing these hypotheses, and in designing more protective ventilation strategies for this patient group.

## Additional file

Additional file 1: Computational model, calibration and additional results. Description of entire computational model, calibration process to fit model to data, fitting results to individual data and dataset from simulation results for. (PDF 2027 kb)

## Abbreviations

ARDS: Acute Respiratory Distress Syndrome
CI: Cardiac Index [ml min^− 1^ m^− 2^]
CO: Cardiac output [ml min^− 1^]
C_RS_: Respiratory System Compliance [ml cmH_2_O^− 1^]
DC: Duty Cycle, inspiration time/ total time for 1 breath
DO_2_: Oxygen delivery [ml min^− 1^]
FIO_2_: Fraction of inspired air constituting of oxygen
Hb: Haemoglobin content in blood [gm l^− 1^]
IE: Inspiration time/ expiration time
LTV: Low Tidal Volume; a lung protective mechanical ventilation strategy
MAP: Mean Arterial Pressure [mm Hg]
MPAP: Mean Pulmonary Arterial pressure [mm Hg]
PaCO_2_: Partial pressure of carbon dioxide in arterial compartment [kpa]
P_alv_: Mean alveolar pressure
PaO_2_: Partial pressure of oxygen in arterial compartment [kpa]
PEEP: Positive End Expiratory Pressure [cm H_2_O]
PF ratio: Ratio of arterial pressure of oxygen to fraction of oxygen in inspired air
pH: Arterial pH value
P_IT_: Intrathoracic pressures
Pplat: Plateau pressure [cm H_2_O]
PvO_2_: Partial pressure of mixed venous oxygen [kpa]
R_aw_: Airway resistance
RQ: Respiratory Quotient
SaO_2_: Arterial oxygen saturation
SvO_2_: Venous oxygen saturation
V_frc_: End Expiratory lung volume at PEEP = 0 cmH_2_O
VILI: Ventilator Induced Lung Injury
VO2: Oxygen consumption [ml min^− 1^]
VQ: Ventilation Perfusion
VR: Respiration rate set by the ventilator [breaths per minute, bpm]
V_T_: Ventilator delivered tidal volume [ml]
ΔP: Airway driving pressure, difference between plateau pressure and total PEEP [cm H_2_O]
