# Supplementary material for: What links ventilator driving pressure with survival in the acute respiratory distress syndrome? A computational study
Source: Respir Res. 2019 Feb 11;20:29. doi: 10.1186/s12931-019-0990-5 (PMC6371576; doi:10.1186/s12931-019-0990-5)
Supplement: Supplementary file 1 — Computational model, calibration and additional results. Description of entire computational model, calibration process to fit model to data, fitting results to individual data and dataset from simulation results for. (PDF 2027 kb) [file 12931_2019_990_MOESM1_ESM.pdf]

What links ventilator driving pressure with survival in the acute respiratory distress syndrome? A computational study.

Online Data Supplement

Anup Das[1], Luigi Camporota[2], Jonathan G. Hardman[3] and Declan G. Bates[1]

Corresponding Author: Prof. Declan G. Bates, School of Engineering, University of Warwick, CV4 7AL, UK, Email: D.Bates@warwick.ac.uk

The online data supplement for this paper contains additional material that could not be included in the main text due to space limitations, and is divided into four sections. The following document describes in detail the simulation model employed in the paper. The optimization strategy used in fitting the model to the ARDS patient data is described. Finally, all simulation results for all individual patients are illustrated.

Contents

Model Description 2

Gas Exchange model 2

Cardiovascular model `8

Model calibration to individual patients 10

Individual matching results of patient data to model 12

Stage 1 results 12

Stage 2 results 13

References for supplementary materials 14

Simulation results for individual patients 17

Model Description

The model employed in this paper has been developed over the past several years and has been applied and validated on a number of different studies [1-8]. The model is organized as a system of several components, each component representing different sections of pulmonary dynamics and blood gas transport, e.g. the transport of air in the mouth, the tidal flow in the airways, the gas exchange in the alveolar compartments and their corresponding capillary compartment, the flow of blood in the arteries, the veins, the cardiovascular compartment, and the gas exchange process in the peripheral tissue compartments. Each component is described as several mass conserving functions and solved as algebraic equations, obtained or approximated from the published literature, experimental data and clinical observations. These equations are solved in series in an iterative manner, so that solving one equation at current time instant (t_k) determines the values of the independent variables in the next equation. At the end of the iteration, the results of the solution of the final equations determine the independent variables of the first equation for the next iteration.

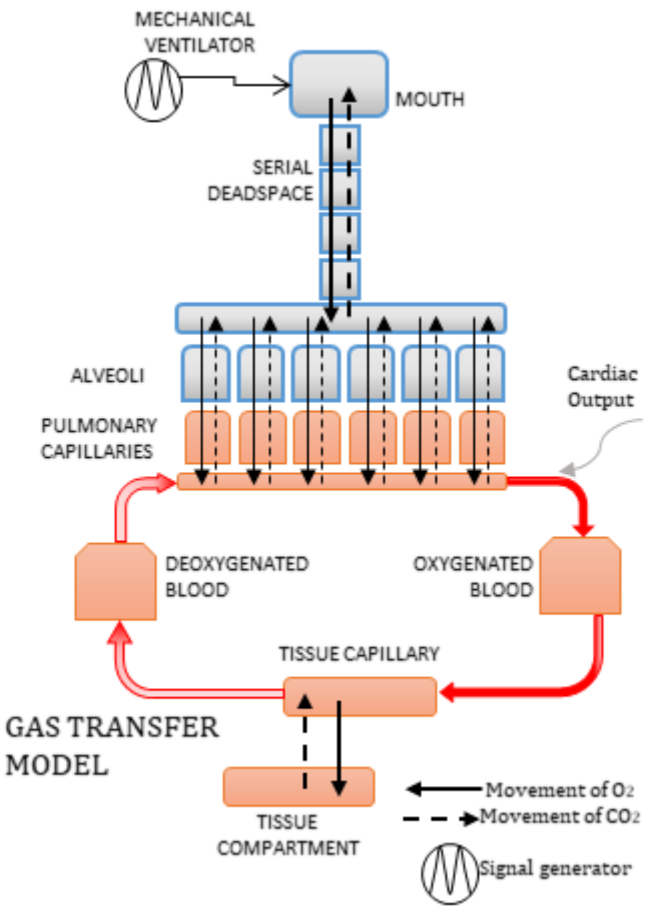

Figure S 1: Schematic of the pulmonary model

The iterative process continues for a predetermined time, *T*, representing the total simulation time, with each iteration representing a 'time slice' *t* of real physiological time (set to 30 ms). At the first iteration ($t_k, k = 0$), an initial set of independent variables are chosen based on values selected by the user. The user can alter these initial variables to investigate the response of the model or to simulate different pathophysiological conditions. Subsequent iterations ($t_k = t_{k-1} + t$) update the model parameters based on the equations below.

The pulmonary model consists of the mechanical ventilation equipment, anatomical and alveolar deadspace, anatomical and alveolar shunts, ventilated alveolar compartments and corresponding perfused capillary compartments. The pressure differential created by the mechanical ventilator drives the flow of gas through the system. The series deadspace (SD) is located between the mouth and the alveolar compartments and consists of the trachea, bronchi and the bronchioles where no gas exchange occurs. Inhaled gases pass through the SD during inspiration and alveolar gases pass through the SD during expiration. In the model, an SD of volume 60ml is split into 50 stacked layers of equal volumes (N_{SD} = 50). No mixing between the compartments of the SD is assumed.

Any residual alveolar air in the SD at the end of expiration is re-inhaled as inspiration is initiated. This residual air is composed of gases exhaled from both perfused alveolar compartments (normal perfusion) and the parallel deadspace (PD) (alveolar compartments with limited perfusion). Therefore, the size of deadspace (SD and PD) can have a significant effect on the gas composition of the alveolar compartments.

The inhaled air is initially assumed to consist of five gases: oxygen (O_2), nitrogen (N_2), carbon dioxide (CO_2), water vapour (H_2O) and a 5th gas (α) used to model additives such as helium or other anaesthetic gases. During an iteration of the model, the flow (*f*) of air to or from an alveolar compartment i at time t_k is determined by the following equation:

$$f_i(t_k) = \frac{(p_v(t_k) - p_i(t_k))}{(R_u + R_{A,i})} \qquad \text{for } i = 1, \dots, N_A \tag{1}$$

where $p_v(t_k)$ is the pressure supplied by the mechanical ventilator at (t_k), $p_i(t_k)$ is the pressure in the alveolar compartment i at (t_k), R_u is the constant upper airway resistance and $R_{A,i}$ is the bronchial inlet resistances of the alveolar compartment i. N_A is the total number of alveolar compartments (for the results in this paper, N_A = 100). The total flow of air entering the SD at time t_k is calculated by

$$f_{SD}(t_k) = \sum_{i=1}^{N_A} f_i(t_k) \tag{2}$$

During the inhaling phase, $f_{SD} \geq 0$, while in the exhaling phase $f_{SD} < 0$.

During gas movement in the SD, the fractions of gases in the layer l of the SD, $F_{l,}$ $(l = 1, \dots, N_{SD})$ is updated based on the composition of the total flow, f_{SD}, and the current composition of $F_{l,}$. If $f_{SD} \geq 0$, then air starts filling from the top layer (l = 1) to the bottom layer ($l = N_{SD}$); and vice versa for $f_{SD} < 0$.

The volume of gas x, in the ith alveolar compartment ($v_{i,x}$), is given by:

$$v_{i,x}(t_k) = \begin{cases} v_{i,x}(t_{k-1}) - f_i(t_k) \cdot \frac{v_{i,x}(t_{k-1})}{v_i(t_k)} & \textit{Exhaling} \\ v_{i,x}(t_{k-1}) + f_i(t_k) \cdot F_{N_{SD}}(t_k) & \textit{Inhaling} \end{cases} \quad \text{for } i = 1, \dots, N_A \tag{3}$$

In (3), x is any of the five gases (O_2, N_2, CO_2, H_2O or α). The total volume of the ith alveolar compartment, v_i is the sum of the volume of the five gases in the compartment.

$$v_i(t_k) = v_{i,O2}(t_k) + v_{i,N2}(t_k) + v_{i,CO2}(t_k) + v_{i,H2O}(t_k) + v_{i,\alpha}(t_k) \tag{4}$$

For the alveolar compartments, the tension at the centre of the alveolus and at the alveolar capillary border is assumed to be equal. The respiratory system has an intrinsic response to low oxygen levels in blood which is to restrict the blood flow in the pulmonary blood vessels, known as Hypoxic Pulmonary Vasoconstriction (HPV). This is modelled as a simple function, resembling the stimulus response curve suggested by Marshall [9], and is incorporated into the simulator to gradually constrict the blood vessels as a response to low alveolar oxygen tension. The atmospheric pressure is fixed at 101.3kPa and the body temperature is fixed at 37.2°C.

At each t_k, equilibration between the alveolar compartment and the corresponding capillary compartment is achieved iteratively by moving small volumes of each gas between the compartments until the partial pressures of these gases differ by <1% across the alveolar-capillary boundary. The process includes the nonlinear movement of O_2 and CO_2 across the alveolar capillary membrane during equilibration.

In blood, the total O_2 content (C_{O2}) is carried in two forms, as a solution and as oxyhaemoglobin (saturated haemoglobin):

$$C_{O2}(t_k) = S_{O2}(t_{k-1}) \cdot Huf \cdot Hb + P_{O2}(t_{k-1}) \cdot O_{2sol} \tag{5}$$

In this equation, S_{O2} is the hemoglobin saturation, *Huf* is the Hufner constant, Hb is the hemoglobin content and O_{2sol} is the O_2 solubility constant. The following pressure-saturation relation, as suggested by [10] to describe the O_2 dissociation curve, is used in this model:

$$S_{O2}(t_k) = \left(\left(\left(P_{O2}^3(t_{k-1}) + 150 \cdot P_{O2}(t_{k-1})\right)^{-1} \times 23400\right) + 1\right)^{-1} \tag{6}$$

S_{O2} is the saturation of the hemoglobin in blood and P_{O2} is the partial pressure of oxygen in the blood. As suggested by [11], P_{O2} has been determined with appropriate correction factors in base excess BE, temperature T and pH (7.5005168 = pressure conversion factor from kPa to mm Hg):

$$P_{O2}(t_k) = 7.5006168 \cdot P_{O2}(t_{k-1}) \cdot 10^{[0.48(pH(t_{k-1})-7.4)-0.024(T-37)-0.0013 \cdot BE]} \tag{7}$$

The CO_2 content of the blood (C_{CO2}) is deduced from the plasma CO_2 content ($C_{CO2plasma}$) [12] by the following equation:

$$C_{CO2}(t_k) = C_{CO2plasma}(t_{k-1}) \cdot \left[1 - \frac{0.0289 \cdot Hb}{(3.352 - 0.456 \cdot S_{O2}(t_k)) \cdot (8.142 - pH(t_{k-1}))}\right] \quad (8)$$

where S_{O2} is the O_2 saturation, Hb is the hemoglobin concentration and pH is the blood pH level. The coefficients were determined as a standardized solution to the McHardy version of Visser's equation [13], by iteratively finding the best fit values to a given set of clinical data. The value of $C_{CO2plasma}$ is deduced using the Henderson-Hasselbach logarithmic equation for plasma C_{CO2} [14] :

$$C_{CO2plasma}(t_k) = 2.226 \cdot s_{CO2} \cdot P_{CO2}(t_{k-1}) \left(1 + 10^{(pH(t_{k-1}) - pK')}\right) \quad (9)$$

where s_{CO2} is the plasma CO_2 solubility coefficient and pK' is the apparent pK (acid dissociation constant of the CO_2 bicarbonate relationship). P_{CO2} is the partial pressure of CO_2 in plasma and '2.226' refers to the conversion factor from miliMoles per liter to ml/100ml. [14] gives the equations for s_{CO2} and pK' as:

$$s_{CO2} = 0.0307 + 0.0057 \cdot (37 - T) + 0.00002 \cdot (37 - T)^2 \quad (10)$$

$$pK' = 6.086 + 0.042 \cdot (7.4 - pH(t_{k-1})) + (38 - T) \cdot\cdot \left(0.00472 + \left(0.00139 - (7.4 - pH(t_{k-1}))\right)\right) \quad (11)$$

$P_{CO2}(t_k)$ is determined by incorporating the standard Henry's law and the s_{CO2} (the CO_2 solubility coefficient above). For pH calculation, the Henderson Hasselbach and the Van Slyke equation [15] are combined. Below is the derivation of the relevant equation. The Henderson-Hasselbach equation (governed by the mass action equation (acid dissociation)) states that:

$$pH = pK + \log\left(\frac{bicarbonate\ concentration}{carbonic\ acid\ concentration}\right) \quad (12)$$

Substituting pK=6.1 (under normal conditions) and the denominator ($0.225 \cdot P_{CO2}$) (acid concentration being a function of CO_2 solubility constant 0.225 and P_{CO2} (in kPa)) gives:

$$pH(t_k) = 6.1 + \log\left(\frac{HCO_3(t_{k-1})}{0.225 \cdot P_{CO2(t_k)}}\right) \quad (13)$$

For a given pH, base excess (BE), and hemoglobin content (Hb), HCO_3 is calculated using the Van-Slyke equation, as given by [15]:

$$HCO_3(t_k) = \left((2.3 \times Hb + 7.7) \times (pH(t_k) - 7.4)\right) + \frac{BE}{(1 - 0.023 \times Hb)} + 24.4 \quad (14)$$

The capillary blood is mixed with arterial blood using the equation below which considers the anatomical shunt (Sh) with the venous blood content of gas x ($C_{v,x}$), the non-shunted blood content from the pulmonary capillaries ($C_{cap,x}$), arterial blood content ($C_{a,x}$), the arterial volume (v_a) and the cardiac output (CO).

$$C_{a,x}(t_k) = \frac{CO(t_k) \cdot (Sh \cdot C_{v,x}(t_k) + (1-Sh) \cdot C_{cap,x}(t_k)) + C_{a,x}(t_k) \cdot (v_a(t_k) - CO(t_k))}{v_a(t_k)} \quad (15)$$

The peripheral tissue model consists of a single tissue compartment, acting between the peripheral capillary and the *active* tissue (undergoing respiration to produce energy). The consumed O_2 (V_{O2}) is removed and the produced CO_2 (V_{CO2}) is added to this tissue compartment. Similarly to alveolar equilibration, peripheral capillary gas partial pressures reach equilibrium with the tissue compartment partial pressures, with respect to the nonlinear movement of O_2 and CO_2. Metabolic production of acids, other than carbonic acid via CO_2 production, is not modeled. After peripheral tissue equilibration of gases, the venous calculations of partial pressures, concentrations and pH calculations are done using comparable equations as above.

A simple equation of renal compensation for acid base disturbance is incorporated. The base excess (BE) of blood under normal conditions is zero. BE increases by 0.1 per time slice if pH falls below 7.36 (to compensate for acidosis) and decreases by 0.1 per time slice if pH rises above 7.4 (under alkalosis).

The simulated patient is assumed to be under complete mechanical ventilation. Consequently, the effects of ventilatory autoregulation by the patient have not been incorporated into the models.

Each alveolar compartment has a unique and configurable alveolar compliance, alveolar inlet resistance, vascular resistance, extrinsic (interstitial) pressure and threshold opening pressure. For the ith compartment of N alveolar compartments, the pressure p_i is determined by:

$$p_i(t_k) = \begin{cases} S_i(v_i(t_k) - V_c)^2 - P_{ext,i} & v_i(t_k) > 0 \\ 0 & \end{cases} \quad \text{for } i = 1, \ldots, N_A \qquad (16)$$

where

$$S_i = k_i {N_A}^2/200000 \quad \text{and} \quad V_c = 0.2V_{FRC}/N_A$$

Equation (18) determines the alveolar pressure p_i (as the pressure above atmospheric in cm H_2O) for the ith compartment of N number of alveolar compartments for the given volume of alveolar compartment, $v_i(t)$ in milliliters. The alveolar compartments are arranged in parallel and interact with the series deadspace with respect to the movement of gases. The flow of air into the alveolar compartments is achieved by a positive pressure provided by the ventilator and the air moves along the pressure gradient. The equation models the behavior of the intact lung / chest-wall complex. The use of the square of the difference between v_i and Vc causes alveolar pressure to increase at volumes below Vc, leading to exhalation and a tendency to "snap shut" (mathematical note: the pressure with respect to volume is thus a U-shaped curve)[16].

P_{ext} (per alveolar unit, in cm H_2O) represents the *effective net pressure* generated by the sum of the effects of factors *outside each alveolus* that act to distend that alveolus; positive components include the outward pull of the chest wall, and negative effects include the compressive effect of interstitial fluid in the alveolar wall. Incorporating P_{ext} in the model allows us to replicate the situation of alveolar units that have less structural support or that have interstitial oedema, and thus have a greater tendency to collapse. A negative value of P_{ext} indicates a scenario where there is compression from outside the alveolus causing collapse. The parameter S_i is a scalar that determines the intra-alveolar pressure for a given volume (with respect to a constant collapsing volume V_c) and is dependent on the parameter k. The units of S_i are cm H_2O ml^{-2}. Finally, V_c is defined as a "constant collapsing volume" at which the alveolus tends to empty (through Laplace effects) and represents a fundamental mechanical property of tissue and surfactant [16]. V_{FRC} is the resting volume of the lung (assumed to be 3 litres).

The effect of the three parameters on the volume–pressure relationship of the alveolar compartments can be observed in the following Figure S2.

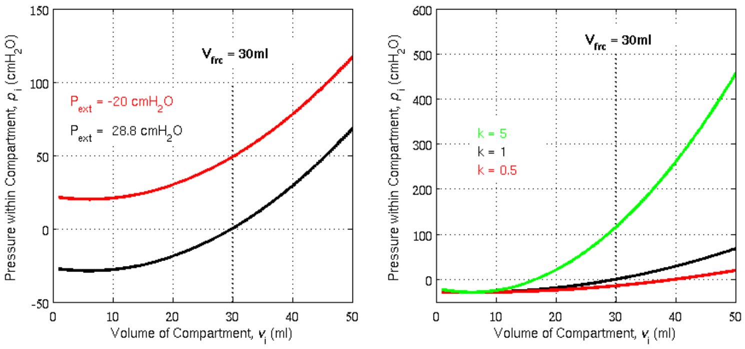

Figure S 2: The effect of varying parameters of Eqn 18 on the pressure volume relationship of the alveolar compartements in the model

For a healthy lung at the end of the expiration, the ventilator pressure would return to zero above atmospheric (resulting in the tracheal pressure also being equal to zero). The nominal values for ($P_{ext,i}$, S_i) have been determined such that at the end of expiration, the alveolar pressure within the compartment is also equal to zero, i.e. at 30 ml, the individual compartments are at rest and consequently the total resting volume of the lung is 3 liters.

We consider each of the three parameters mentioned above ($P_{ext,i}$, S_i) to be different yet essential components for representing a diseased lung, that affect the volume pressure relationship of the alveolar compartments. For

example, for a given volume v_i, increasing S_i increases the corresponding alveolar pressure of the alveolar compartment. When compared to another compartment with a lower S_i, a larger pressure from the mechanical ventilator would be needed to drive air into the compartment; thus effectively the compartment will be behaving as a stiffer lung unit.

Decreasing $P_{ext,i}$ increases the alveolar pressure such that the pressure gradient (especially during exhaling) forces the air out of the alveolar compartment until the volume of the compartment collapses (v_i = 0 ml). Note that, in effect, the parameters are influencing the resting volume of the compartments (when the alveolar pressure, p_i, is equal to zero). If p_i< 0 cm H_2O, the pressure gradient will cause the flow into the alveolar compartment (as ventilator pressure will always be ≥ 0 cm H_2O) until p_i reaches 0 cm H_2O.

In the model, the airway resistance $R_{aw}$is determined by the following equation for N parallel compartments:

$$\frac{1}{R_{aw}} = \frac{1}{R_{B,1}} + \frac{1}{R_{B,2}} + \cdots + \frac{1}{R_{B,N_A}}, \text{ for } i = 1, \ldots, N_A \tag{17}$$

where $R_{B,i}$ is the bronchial inlet resistance of the ith compartment, which is defined by:

$R_{B,i} = m_i R_{B0}$

where R_{B0} corresponds to the default bronchial inlet resistance of an alveolar compartment. R_{B0} is set to $1 \times 10^{-5} \cdot$ N (the inlet resistance is higher for a model with more compartments as the volume of each compartment decreases) for a healthy lung, giving a resistance of 0.001 kpa per ml per minute for 100 compartments. m_i is a coefficient of the airway resistance, representing a dynamic change in airway resistance and is determined by the equation:

$$m_i = \begin{cases} 1, & \mathrm{t}_{\mathrm{o},i} \leq 0 \\ 10^{10}, & \mathrm{t}_{\mathrm{o},i} > 0 \end{cases} \quad \text{for } i = 1, \ldots, N_A \tag{18}$$

where,

$$\mathrm{t}_{\mathrm{o},i} = \begin{cases} \mathrm{t}_{\mathrm{o},i} - t, & p_{trachea} \geq \mathrm{TOP}_i \\ \tau_{c,i}, & p_{trachea} < \mathrm{TOP}_i \end{cases} \quad \text{for } i = 1, \ldots, N_A \tag{19}$$

$p_{trachea}$ is the pressure in the trachea and TOP_i is a value between 5 and 50 cm H_2O for the ith alveolar compartment. Additionally, a threshold opening pressure (TOP) at low lung volumes needs to be attained for a collapsed alveolar unit to open. Recruitment is a time dependent process, with different airways recruiting at different times, once the threshold pressure has been achieved [17, 18]. The equations within the model are solved iteratively as a discretized system. Each iteration represents a physiological time slice of *t* (10 ms). The time dependant recruitment phenomenon is achieved in the model by the introduction of a parameter $\mathrm{t_o}$. For collapsed compartments, $\mathrm{t_o}$ is set to τ_c which represents the time it could take for collapsed alveoli to open after a threshold pressure is reached. Once $p_{trachea} \geq \mathrm{TOP_i}$ is satisfied, the counter $\mathrm{t_o}$ decrements during every iteration, and triggers the opening of the airway (m_i= 1) as $\mathrm{t_o} \leq 0$. Otherwise m_i is set to a high value (10^{10}) to represent a collapsed airway. We based the range of values for TOP used in these simulations on the work done by Crotti and collaborator [19].

N_A (the number of alveolar compartments) is fixed and set by the user (i.e. they do not change during a simulation). Therefore, during a simulation, m_i, chiefly represents the relatively small changes in inlet resistance during tidal ventilation. Furthermore, R_{B0} are also preset and fixed, and do not change during the simulation. The only change in airway resistance which is dynamic is m_i which is dependent on the volume v_i at time($\mathrm{t_k}$).

Finally, the pulmonary vascular resistance PVR is determined by

$$\frac{1}{PVR} = \frac{1}{R_{V,1}} + \frac{1}{R_{V,2}} + \cdots + \frac{1}{R_{V,N_A}}, \text{ for } i = 1, \ldots, N_A \tag{20}$$

where the resistance for each compartment $R_{V,i}$ is defined as

$$R_{V,i} = \delta_{Vi} R_{V0} \tag{21}$$

R_{V0} is the default vascular resistance for the compartment with a value of $160 \cdot N_A$ dynes s cm^{-5} min^{-1}, and δ_{Vi} is the vascular resistance coefficient, used to implement the effect of Hypoxic Pulmonary Vasoconstriction.

The net effect of these components of the simulation is that the defining, clinical features of ARDS may be observed in the model: alveolar gas-trapping (with intrinsic PEEP), collapse-reopening of alveoli (with gradual reabsorption of trapped gas if re-opening does not occur), limitation of expiratory flow etc.

The cardiovascular model consists of 19 compartments. Each compartment x, is described with a pressure P_x, a volume V_x and a flow leaving the compartmentF_x, which are iteratively updated in a sampling interval. Furthermore, each compartment has the following fixed parameters: a resistance R_x to the flow out of the compartment reflecting the viscosity of the compartment, a coefficient λ_x governing the elastance of each compartment, a coefficient $P_{x,c}$ and $V_{x,u}$, depicting the unstressed volume of the compartment. The ventricles are modeled as having time varying elastances over the duration of a cardiac cycle using different exponential functions to describe the filling and emptying of the ventricles [20]. The shift from the systolic to diastolic relationship is governed by a pulsating activation function with period$T$. For all the compartments, vascular elastance is assumed to be nonlinear and to have an exponential relationship governed by the following equation,

$$P_x = P_{x,c} e^{\frac{\lambda_x (V_x - V_{x,u})}{(V_x + V_{x,u})}}$$

where the subscript x represents the compartment number and the $\lambda_x, P_{x,c}, V_{x,u}$ are constants that give flexibility in fitting specific shapes and peaks of pressure waveforms that could be observed from clinical data. The model

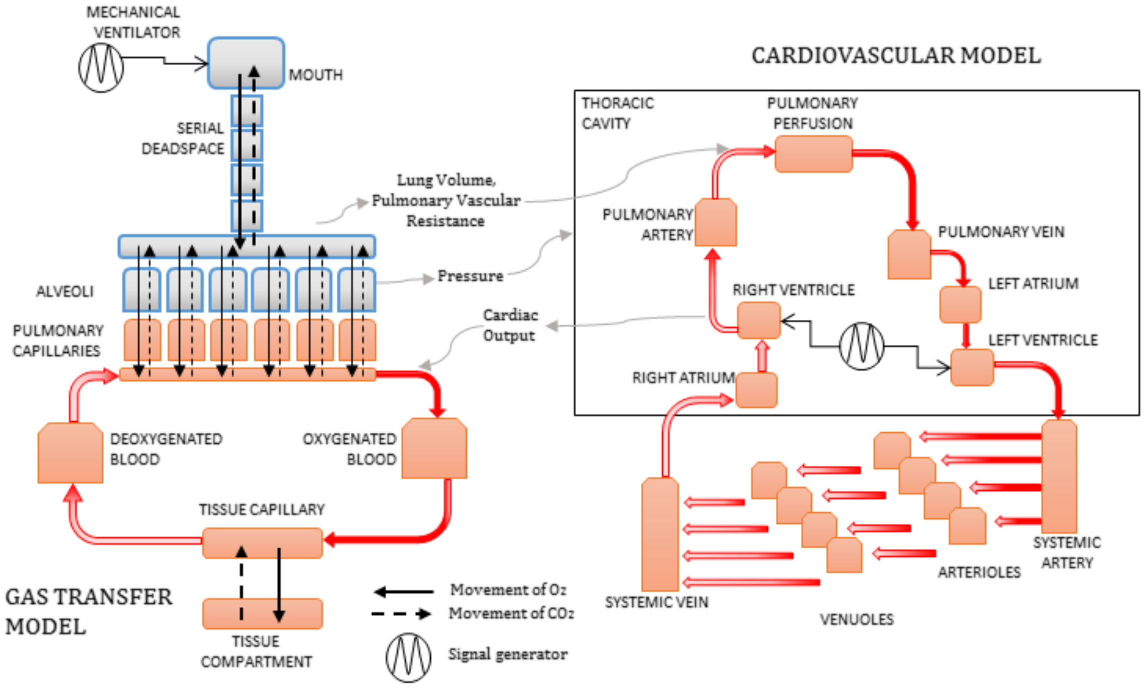

Figure S 3: Schematic of the cardiovascular model with respect to the original pulmonary (gas transfer model)

employs separate pressure volume relationships for the systolic and diastolic behavior of ventricles. The left ventricular pressure calculation is given by:

$$P_{lv} = \varphi P_{lv,sys,c} e^{\frac{\lambda_{lv.sys}(V_{lv} - V_{lv,sys,u})}{(V_{lv} + V_{lv,sys,u})}} + (1-\varphi) P_{lv,dys,c} e^{\frac{\lambda_{lv.dys}(V_{lv} - V_{lv,dys,u})}{(V_{lv} + V_{lv,dys,u})}} \quad 3$$

The right ventricular pressure calculation is given by:

$$P_{rv} = \varphi P_{rv,sys,c} e^{\frac{\lambda_{rv.sys}(V_{rv} - V_{rv,sys,u})}{(V_{rv} + V_{rv,sys,u})}} + (1-\varphi) P_{lv,dys,c} e^{\frac{\lambda_{rv.dys}(V_{rv} - V_{rv,dys,u})}{(V_{rv} + V_{rv,dys,u})}}$$

(24)

The function φ is a ventricle activation function which is assumed to attain the maximum value of $\varphi = 1$ at the peak of systolic contraction. The function φ attains its minimum value 0 at maximal diastole relaxation. A squared half-sine wave function [20, 21] is adopted for φ given by:

$$\varphi = \begin{cases} \left(\sin\left(\pi T \dfrac{u}{T_{sys}}\right)\right)^2 & if\ u \geq 0\ and\ u \leq \dfrac{T_{sys}}{2} \\ 0, & if\ u > \dfrac{T_{sys}}{2}\ and\ u \leq 1 \end{cases}$$

$$T = 1/HR, \quad T_{sys} = \frac{(T_{sys,o} - k_{sys})}{T},$$

$$where\ T_{sys,o} = 0.5\ and\ k_{sys} = 0.075$$

□□5□

□□6□

u is a real number ranging between 0 and 1 and it models the fraction of the cardiac cycle. $u = 0$ at the end of systole and $u = 1$ at the end of diastole. T_{sys} indicates the systolic period which is proportional to the heart rate HR (in seconds).

The blood flow between compartments is determined by the pressure gradient between compartments across a linear time invariant resistance R_x.

$$F_x = \frac{\eta_x (P_x - P_y)}{R_x},$$ (27)

$$\eta_x = \begin{cases} 0, & if\ P_x < P_y \\ 1, & if\ P_x \geq P_y \end{cases}.$$

□28□

The parameter η_x allows the blood to flow in one direction but can be altered to investigate flow backwards into a compartment, such as during aortic regurgitation. The volume of the blood in each compartment is computed by applying conservation of mass as follows

$$V_x = (V_{x0} + (F_{\bar{x}} - F_x)\Delta t),$$ (29)

where $F_{\bar{x}}$ is the flow entering the xth compartment (i.e. the flow leaving the upstream compartment) and F_x is the flow leaving the compartment x. V_{x0} is the volume of compartment x before the iteration, and Δt is the size of the time period in this iteration (set to 1 ms). The total amount of blood in the whole body is obtained by $V_T = \sum V_x$.

The model includes the effect of radial compressive and axial stretching forces exerted onto pulmonary capillaries as a result of increase in lung volume and pressure. The overall effect on resistance to flow through each capillary is difficult to quantify, but we assume the following: (i) at alveolar volumes above the functional residual capacity (FRC), the vessels become compressed and raise the pulmonary vascular resistance (PVR), (ii) at alveolar volumes below FRC, the vessels can collapse and thus result in an increase in PVR, while closer to FRC the PVR remains unaffected. A separate mechanism called hypoxic vasoconstriction, of the vessels contracting in response to hypoxia as a result of alveolar collapse, is already present in the existing pulmonary model. The resultant 'U' shape change in PVR at around the FRC has been suggested previously [22] and has been implemented in this model as follows. The pulmonary vascular resistance PVR is determined as given in equation 23, but the vascular resistance for each alveolar compartment, $R_{V,i}$, is defined has been modified from (24) to

$$R_{V,i} = pvr_{mult,i}\delta_{Vi}R_{V0}. \quad \text{for } i = 1,2, \dots, N_A, \tag{30}$$

N_A is the number of alveolar compartments (set to 100), R_{V0} is the default vascular resistance for the compartment with a value set to $160 \cdot N_A$ dynes s cm^{-5} min^{-1}, and δ_{Vi} is the vascular resistance coefficient, used to implement the effect of Hypoxic Pulmonary Vasoconstriction (and to increase vascular resistance). $pvr_{mult,i}$ is calculated as follows:

$$pvr_{mult,i} = \left(\left(1 + 0.5\left(\frac{(v_i - v_{FRC})}{v_{FRC}}\right)^2\right)\left(1 + \frac{p_i}{q_{pvr}}\right)\right)^{n_{pvr}}, \tag{31}$$

where, p_i is the pressure generated within the ith alveolar compartment, v_i is the volume of the ith alveolar compartment, v_{FRC} is a constant representing the volume of the alveolar compartment at rest (fixed to 30 ml). n_{pvr} and q_{pvr} is used to adjust the effect on pulmonary vascular resistance. n_{pvr} is set to 1 and q_{pvr} has been set to 30 but they can be modified to fit patient data.

Increase in lung pressures, such as those observed during the addition of incremental PEEP (Positive End Expiratory Pressure) during ventilation, serves to increase mean intrathoracic pressure to bring about the recruitment and maintenance of collapsed alveolar lung units as mentioned above. In addition to the effect on PVR, the average alveolar compartment pressure within the lung exerts an extrinsic pressure which is applied to the intra-thoracic vascular compartments. This phenomenon is known as splinting. The pressure calculation of the compartments within the thoracic cavity therefore has an additional term, P_{tp}, added to them, representing the intrathoracic pressure

$$P_{tp} = \gamma_{pvr}(P_{lungs} - P_{atm}).$$
(□2)

$\gamma_{pvr} = 0$ for extra-thoracic compartments. Within the thoracic cavity a range of values (0.1-0.8) is used for γ_{pvr} to fit patient data.

Model parameters have been identified from the published literature where available – parameters whose values were not available from the literature were adjusted within their physiological ranges based on the resulting pressure and flow waveforms.

Model Calibration to individual patients

The complete model calibration to disease configuration algorithm is shown in Table S1.

Selection of Patient Data
Data for 25 ARDS patients was extracted from Borges 2006, selected due to their inclusion of data on hemodynamic responses in ARDS patients to changes in mechanical ventilation, specifically changes in cardiac output to variation in positive end expiratory pressure (PEEP). The patients represent a cross section of ARDS patients, with varying severity (based on the Berlin definition) and cardiac volemic status. Baseline patient information is listed in the main manuscript Table 1.

Table S1: Algorithm for fitting model outputs to patient data in the integrated model

Select model parameters for fitting.<br>Select model parameters for the pulmonary model<br>Select model parameters for the cardiovascular model through sensitivity analysis.

STAGE – 1: Determine model parameters *x* for pulmonary model<br>Use the pulmonary model, global optimization, and<br>Eq. (34) to determine parameter values (*x*)<br>STAGE - 2: Determine model parameters for cardiovascular model<br>Use the integrated cardiopulmonary model, global<br>optimization, and Eq. (35), to determine parameter values<br>(*u*) that minimize E_2 at different values of PEEP

Model parameter configuration using optimization

The model was calibrated against the dataset from Borges et al. 2006 as follows. Pulmonary model parameters were identified at PEEP 10 cmH_2O that matched the model outputs (PF ratio, arterial carbon dioxide tension, mixed venous oxygen saturation (SvO_2), and static compliance [Cstat]) to baseline data given in Borges et al. [14]. All model parameters were constrained to vary between appropriate physiological ranges.

The key model parameters (x) to be optimized for each of the 100 alveolar compartments were P_{ext}, k_{stiff} and TOP, representing the extrinsic pressure acting on an alveolar compartment, the stiffness of the compartment and the threshold opening pressure, respectively. The values for respiratory quotient (RQ), rate of breathing (VR), total oxygen consumption (VO_2), hemoglobin levels (Hb), and the inspiratory duty cycle were additional parameters determined by the optimization algorithm. In this case, the model-fitting problem was formulated to search for a configuration of model parameter values (*x*) that minimizes objective function E_1 in the equation below:

$$\min_x E_1 = \sqrt[2]{\sum_{i=1}^{4} r_j^2} \quad \text{where } r_i = \frac{y_i - y_i'}{y_i'} \qquad (34)$$

where y = [PaO_2, $PaCO_2$, TOP_{mean}, P_{peak}] are the model outputs and y' = [PaO_2', $PvCO_2$', TOP_{mean}', P_{peak}'] are the target values. PaO_2' and $PaCO_2$' are measurements obtained from the patient data. TOP_{mean} is the average TOP of the alveolar units, which is set to 20 cmH_2O [19]. P_{peak} is the peak airway pressure which is minimized to 30 cm H_2O (a target in the 2000 ARDSnet report [30]).

Stage 2 of the fitting process required a search for the optimal values of the parameters (*u*) of the cardiovascular models (*Figure S2*) effectively allowing the modification of the cardiovascular function. The optimization process was used to fit the data for changes in CO, $PaCO_2$, SvO_2 and mean arterial pressure (MAP) to changes in PEEP. For this stage, the optimization problem was formulated to find a configuration of model parameters (*u*) that minimizes the objective function E_2:

$$\min_u E_2 = \sqrt[2]{\sum_{j=1}^{k} r_j^2} \quad \text{where } r_j = \frac{y_j - y_j'}{y_j'} \qquad (35)$$

where y_j = [CO_i, $PaCO2_j$, $SvO2_j$ MAP_j] are the model outputs and y'_j= [CO_i', $PaCO2_i$', $SvO2_i$' MAP_i'] are the CO, $PaCO_2$, SvO_2 and MAP values reported in the data for the jth PEEP value, with k different settings of PEEP.

Genetic algorithms (GA's) were employed for the optimization processes of Stage 1 and 2, primarily due to their ease of application in problems with large and small parameter search spaces, and their capability to converge to the global optimum even in highly non-convex parameter spaces. Initial model calibration and analysis were performed on a 64-bit Intel Core i7 3.7 GHz PC, running Matlab (R2015a). Model calibration to data was performed using the 'Minerva' high performance computing cluster provided by the University of Warwick (396 nodes, each with 2×hexa-core 2.66 GHz 24 GB RAM) running Matlab (2015a) with global optimization and parallel computing toolboxes.

Figure S4 displays the results of stage 2, where the model outputs were matched to increments in PEEP.

Individual matching results of patient data to model

Table S2: Model calibration Stage 1 – Results:ß

Pid	PF (data)	PF (model)	Cstat (data)	Cstat (model)	PaCO2 (kpa)	Resp. rate	Duty Cycle	PEEP (cmH2O)	SaO2	SvO2	shunt	pH	Hb	VO2 (ml/min)	RQ	VCO2 (ml/min)
1	111	109.9	24	23.7	8.2	12.1	0.4	10.0	1.0	0.7	0.3	7.3	120.0	308.2	0.9	269.4
2	167	172.2	22	21.7	8.7	14.1	0.4	10.0	1.0	0.8	0.3	7.3	123.2	304.8	0.9	263.4
3	269	267.3	37	26.5	8.7	13.2	0.4	10.0	1.0	0.9	0.1	7.3	116.5	309.4	0.9	272.0
4	45	55.3	13	16.1	6.9	13.0	0.4	10.0	0.9	0.6	0.5	7.3	113.1	303.4	0.8	240.3
5	66	70.4	11	16.2	8.0	12.1	0.4	10.0	0.9	0.7	0.5	7.3	117.9	298.7	0.9	259.4
6	55	66.8	29	21.1	6.9	12.1	0.4	10.0	0.9	0.6	0.4	7.3	112.2	307.5	0.8	239.5
7	59	68.0	29	19.5	7.5	13.1	0.4	10.0	0.9	0.6	0.4	7.3	119.4	308.1	0.8	245.7
8	48	62.6	23	17.9	6.6	14.0	0.4	10.0	0.9	0.6	0.4	7.4	113.9	308.6	0.8	242.2
9	83	85.7	23	20.5	7.9	14.1	0.4	10.0	1.0	0.7	0.4	7.3	128.5	307.4	0.8	256.9
10	61	72.0	35	20.8	7.1	12.2	0.4	10.0	0.9	0.7	0.4	7.3	115.8	302.6	0.8	250.2
11	184	189.6	31.2	21.3	9.0	14.6	0.4	10.0	1.0	0.9	0.2	7.3	124.6	304.3	0.9	272.5
12	78	83.1	26.7	22.0	8.4	12.1	0.4	10.0	0.9	0.7	0.4	7.3	111.5	307.3	0.8	237.5
13	69	73.8	22.7	21.1	8.3	12.1	0.4	10.0	0.9	0.7	0.4	7.3	110.1	308.8	0.8	232.2
14	208	207.3	17	20.8	8.6	12.0	0.4	10.0	1.0	0.8	0.3	7.3	114.4	270.4	0.9	238.2
15	294	291.3	37.5	28.6	8.8	12.0	0.4	10.0	1.0	0.9	0.1	7.3	121.8	271.9	0.9	242.3
16	130	131.3	20.3	22.5	8.7	12.1	0.4	10.0	1.0	0.8	0.3	7.3	120.8	306.3	0.8	230.2
17	105	107.0	37.2	23.4	8.6	12.0	0.4	10.0	1.0	0.7	0.4	7.3	112.9	305.3	0.8	252.4
18	191	194.0	32.5	24.8	8.9	12.0	0.4	10.0	1.0	0.8	0.3	7.3	120.2	307.0	0.9	273.8
19	61	73.1	31.6	20.5	8.0	12.3	0.4	10.0	0.9	0.7	0.4	7.3	116.5	306.9	0.7	219.2
20	206	205.5	66.7	23.9	9.2	12.1	0.4	10.0	1.0	0.8	0.3	7.3	113.0	302.9	0.9	260.9
21	81	87.4	27.3	22.7	8.2	12.0	0.4	10.0	1.0	0.7	0.4	7.3	119.5	307.2	0.8	259.8
22	69	74.6	23.2	21.4	7.8	12.1	0.4	10.0	0.9	0.7	0.4	7.3	117.3	304.0	0.8	250.5
23	263	270.8	38.2	28.3	8.5	12.4	0.4	10.0	1.0	0.9	0.1	7.3	115.6	301.8	0.8	251.4
24	212	222.3	33.6	26.8	8.8	12.1	0.4	10.0	1.0	0.9	0.2	7.3	113.0	304.0	0.8	247.5
25	161	166.4	35.1	24.0	8.4	12.1	0.4	10.0	1.0	0.8	0.3	7.3	111.3	306.5	0.8	231.0

Stage 2 – Results:

The figure below shows the individual patient calibration results from Stage 2.

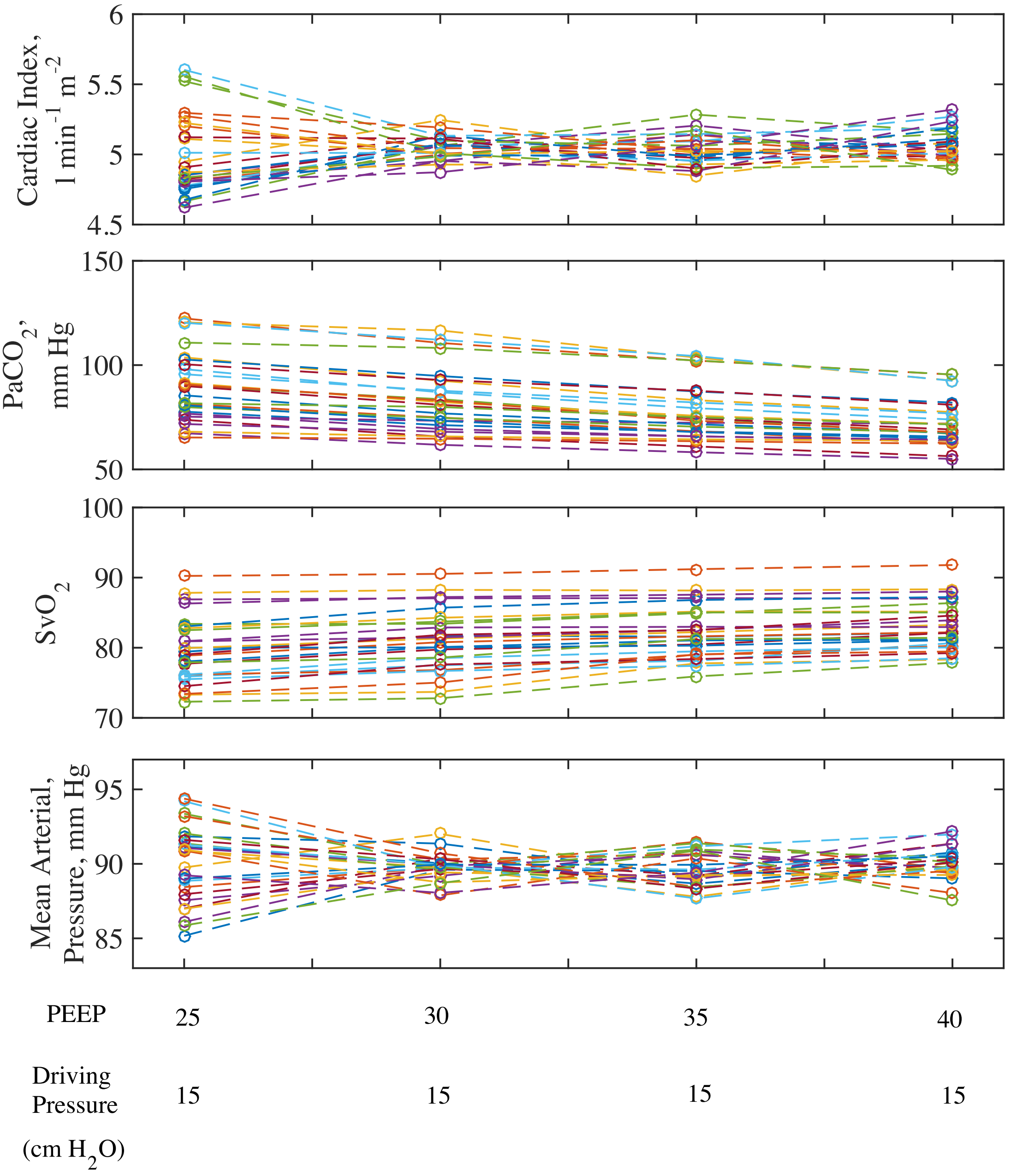

Figure S 4: Result of fitting the CVS model (Stage 2 of model calibration process)

References for Supplementary materials

1. Hardman J, Aitkenhead A: Estimation of alveolar deadspace fraction using arterial and end-tidal CO2: a factor analysis using a physiological simulation. *Anaesthesia and intensive care* 1999, 27(5):452.

2. Hardman J, Bedforth N: Estimating venous admixture using a physiological simulator. *British journal of anaesthesia* 1999, 82(3):346-349.

3. Hardman J, Bedforth N, Ahmed A, Mahajan R, Aitkenhead A: A physiology simulator: validation of its respiratory components and its ability to predict the patient's response to changes in mechanical ventilation. *British journal of anaesthesia* 1998, 81(3):327-332.

4. Hardman JG, Aitkenhead AR: Validation of an original mathematical model of CO2 elimination and dead space ventilation. *Anesthesia & Analgesia* 2003, 97(6):1840-1845.

5. Hardman J, Wills J: The development of hypoxaemia during apnoea in children: a computational modelling investigation. *British journal of anaesthesia* 2006, 97(4):564-570.

6. Das A, Gao Z, Menon P, Hardman J, Bates D: A systems engineering approach to validation of a pulmonary physiology simulator for clinical applications. *Journal of The Royal Society Interface* 2011, 8(54):44-55.

7. McCahon R, Columb M, Mahajan R, Hardman J: Validation and application of a high-fidelity, computational model of acute respiratory distress syndrome to the examination of the indices of oxygenation at constant lung-state. *British journal of anaesthesia* 2008, 101(3):358-365.

8. Hardman JG, Al-Otaibi HM: Prediction of arterial oxygen tension: validation of a novel formula. *American journal of respiratory and critical care medicine* 2010, 182(3):435-436.

9. Marshall BE, Clarke WR, Costarino AT, Chen L, Miller F, Marshall C: The dose-response relationship for hypoxic pulmonary vasoconstriction. *Respir Physiol* 1994, 96(2-3):231-247.

10. Severinghaus JW: Simple, accurate equations for human blood O2 dissociation computations. *J Appl Physiol Respir Environ Exerc Physiol* 1979, 46(3):599-602.

11. Severinghaus JW: Blood gas calculator. *J Appl Physiol* 1966, 21(3):1108-1116.

12. Douglas AR, Jones NL, Reed JW: Calculation of whole blood CO2 content. *J Appl Physiol (1985)* 1988, 65(1):473-477.

13. McHardy GJ: The relationship between the differences in pressure and content of carbon dioxide in arterial and venous blood. *Clin Sci* 1967, 32(2):299-309.

14. Kelman GR, Nunn JF: Nomograms for correction of blood Po2, Pco2, pH, and base excess for time and temperature. *J Appl Physiol* 1966, 21(5):1484-1490.

15. Siggaard-Andersen O: The van Slyke equation. *Scand J Clin Lab Invest Suppl* 1977, 146:15-20.

16. Lachmann B: Open up the lung and keep the lung open. *Intensive Care Med* 1992, 18(6):319-321.

17. Hickling KG: The pressure-volume curve is greatly modified by recruitment. A mathematical model of ARDS lungs. *Am J Respir Crit Care Med* 1998, 158(1):194-202.

18. Bates JH, Irvin CG: Time dependence of recruitment and derecruitment in the lung: a theoretical model. *J Appl Physiol (1985)* 2002, 93(2):705-713.

19. Crotti S, Mascheroni D, Caironi P, Pelosi P, Ronzoni G, Mondino M, Marini JJ, Gattinoni L: Recruitment and derecruitment during acute respiratory failure: a clinical study. *Am J Respir Crit Care Med* 2001, 164(1):131-140.

20. Ursino M: Interaction between carotid baroregulation and the pulsating heart: a mathematical model. *American Journal of Physiology-Heart and Circulatory Physiology* 1998, 275(5):H1733-H1747.

21. Piene H: Impedance matching between ventricle and load. *Annals of biomedical engineering* 1984, 12(2):191-207.

22. Shekerdemian L, Bohn D: Cardiovascular effects of mechanical ventilation. *Archives of disease in childhood* 1999, 80(5):475-480.

Simulation Results individual Patients

Patient 1.

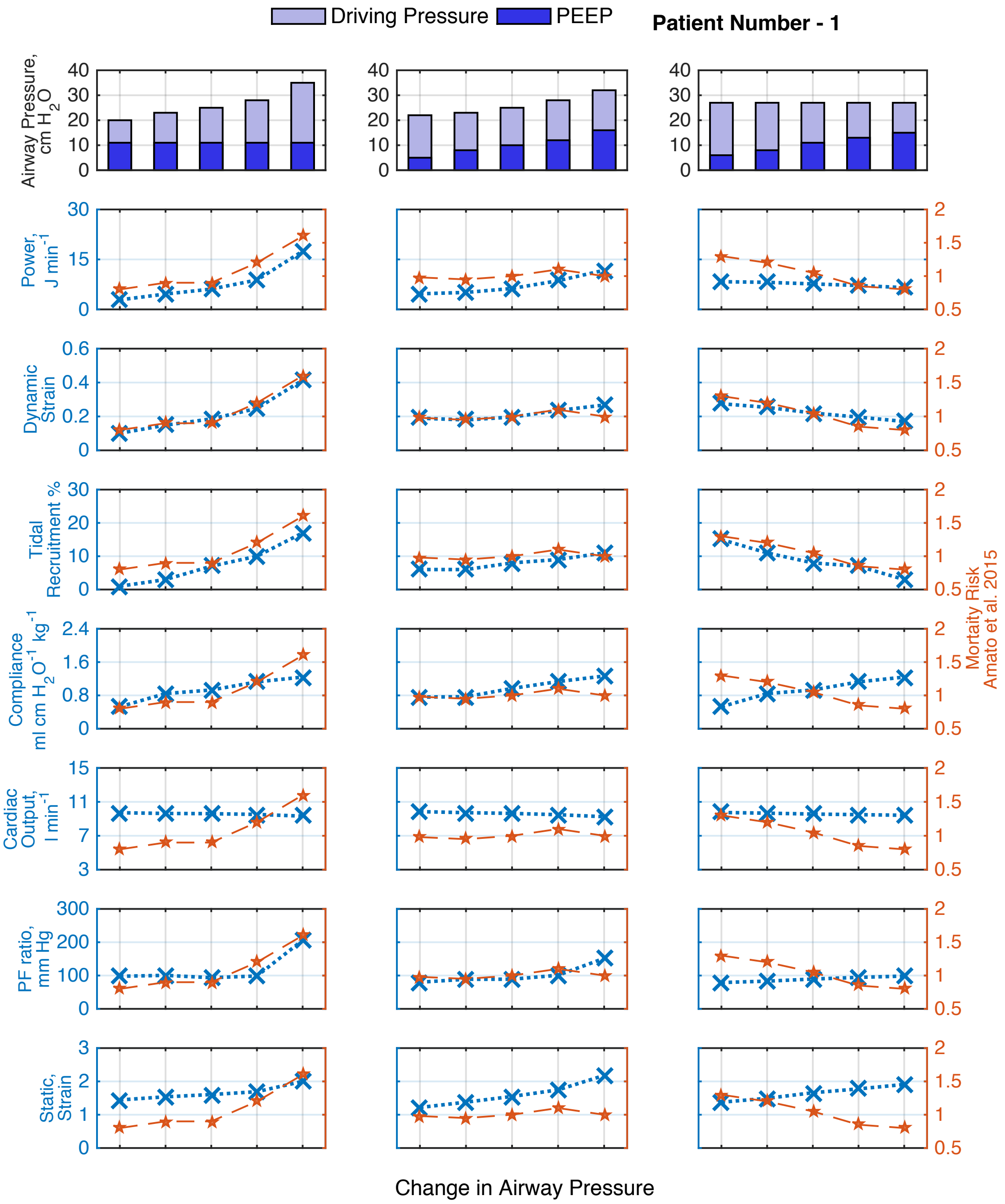

Patient 2.

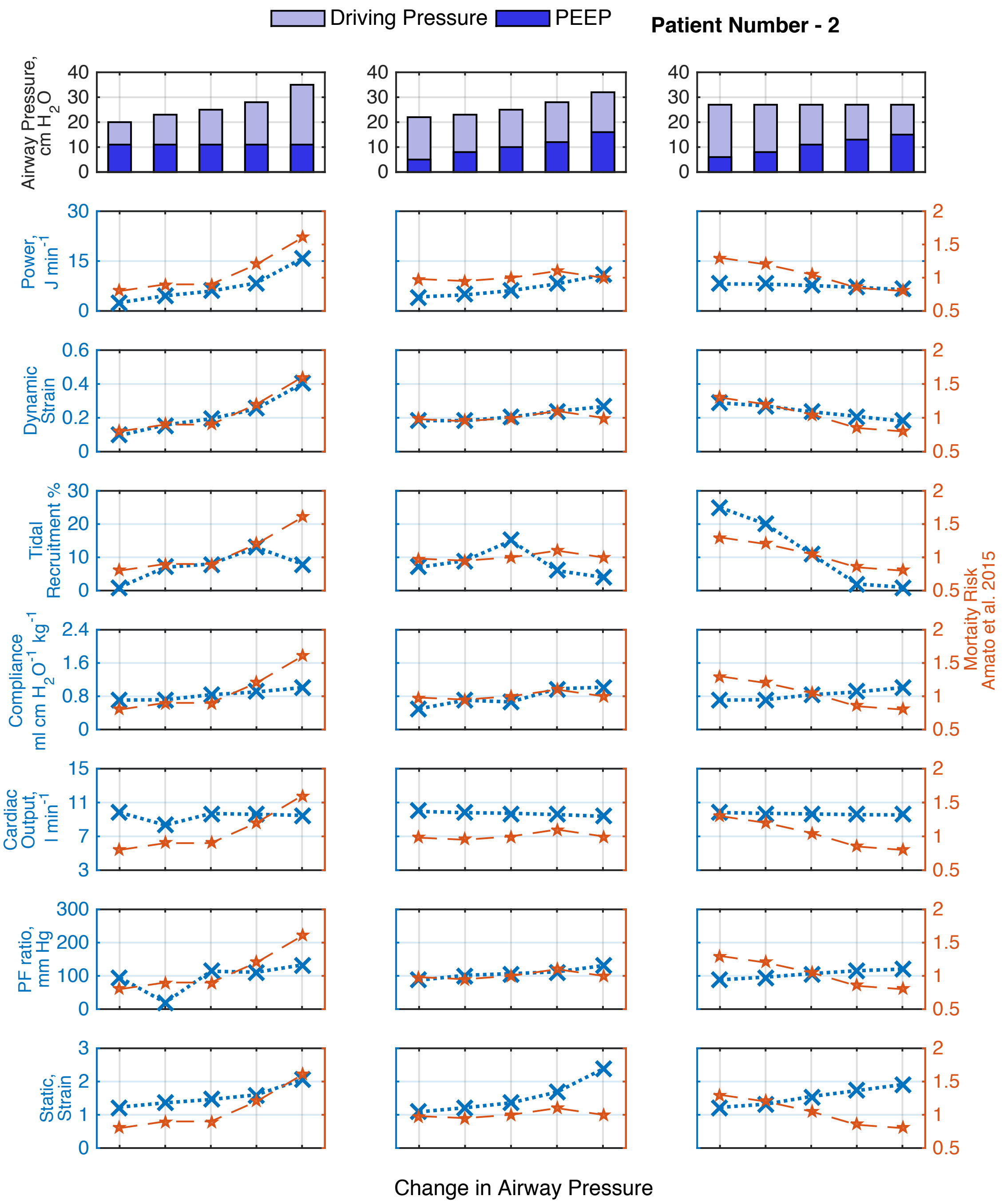
Driving Pressure
PEEP
Patient Number - 2
Airway Pressure, cm H_2O
Power, J min^{-1}
Dynamic Strain
Tidal Recruitment %
Compliance ml cm H_2O^{-1} kg^{-1}
Cardiac Output, l min^{-1}
PF ratio, mm Hg
Static, Strain
Mortaity Risk
Amato et al. 2015
Change in Airway Pressure

Patient 3.

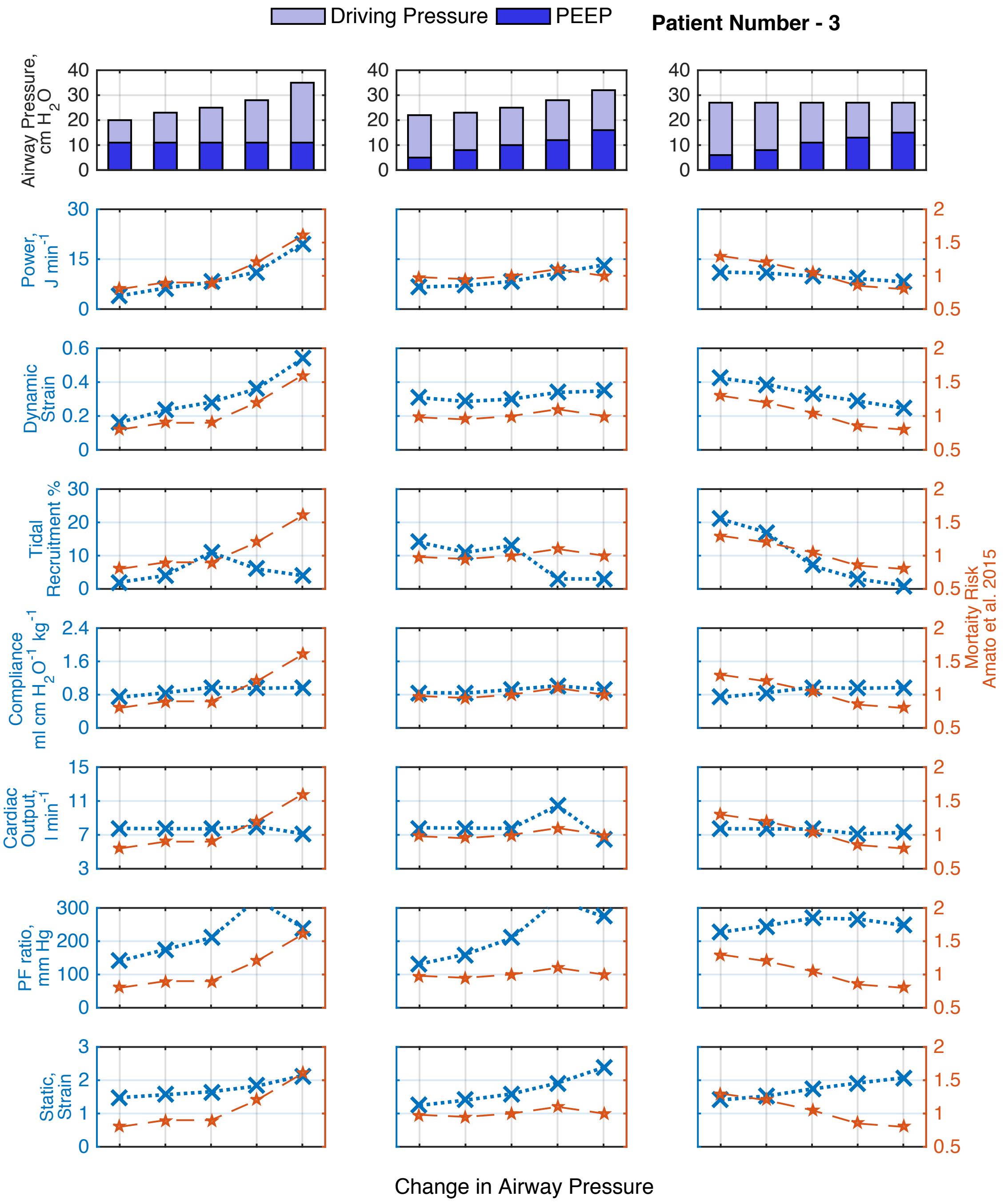
Driving Pressure
PEEP
Patient Number - 3
Airway Pressure, cm H_2O
Power, J min^{-1}
Dynamic Strain
Tidal Recruitment %
Compliance ml cm H_2O^{-1} kg^{-1}
Cardiac Output, l min^{-1}
PF ratio, mm Hg
Static, Strain
Mortality Risk Amato et al. 2015
Change in Airway Pressure

Patient 4.

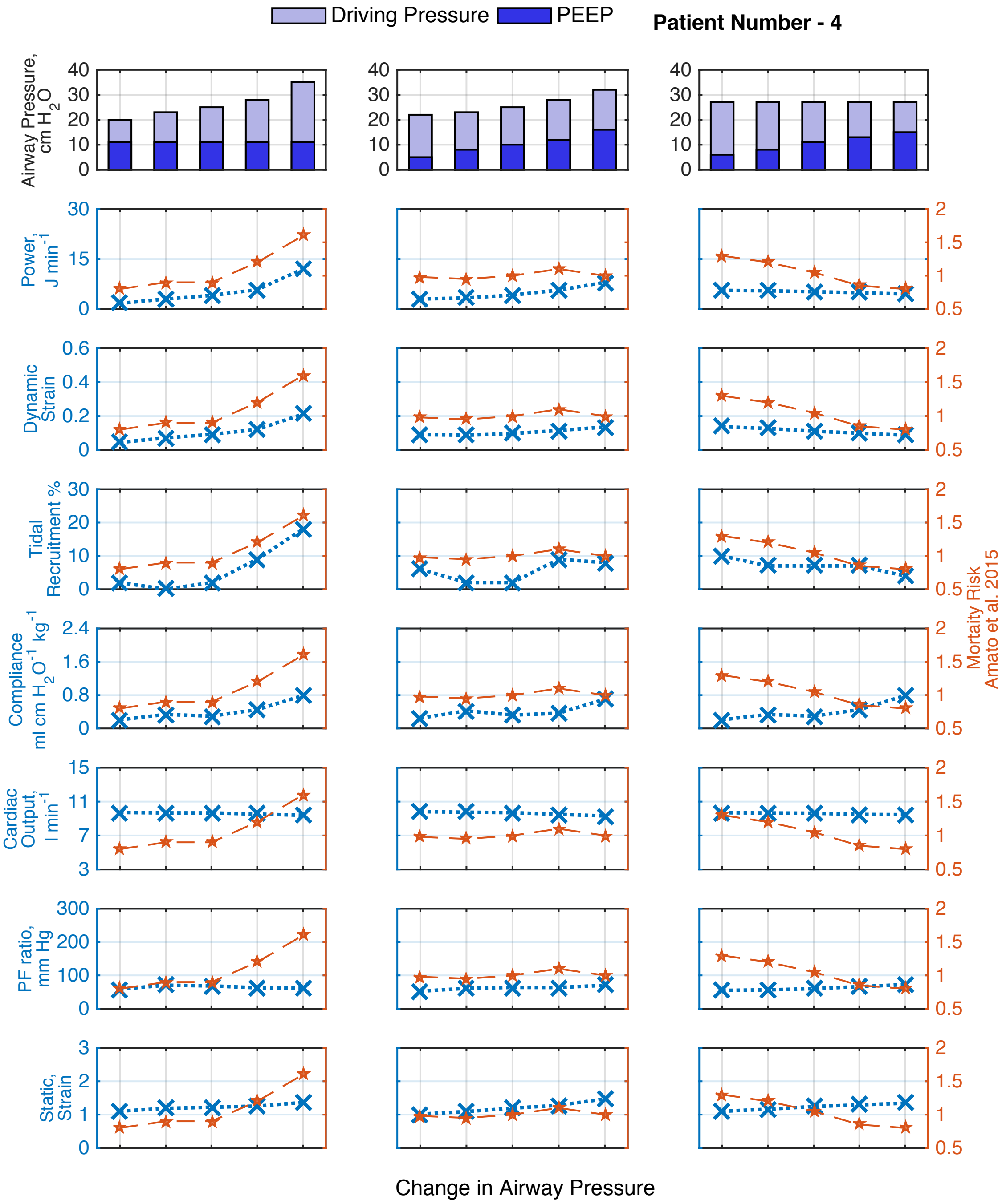
Driving Pressure
PEEP
Patient Number - 4
Airway Pressure, cm H_2O
Power, J min^{-1}
Dynamic Strain
Tidal Recruitment %
Compliance ml cm H_2O^{-1} kg^{-1}
Cardiac Output, l min^{-1}
PF ratio, mm Hg
Static, Strain
Mortaity Risk
Amato et al. 2015
Change in Airway Pressure

Patient 5.

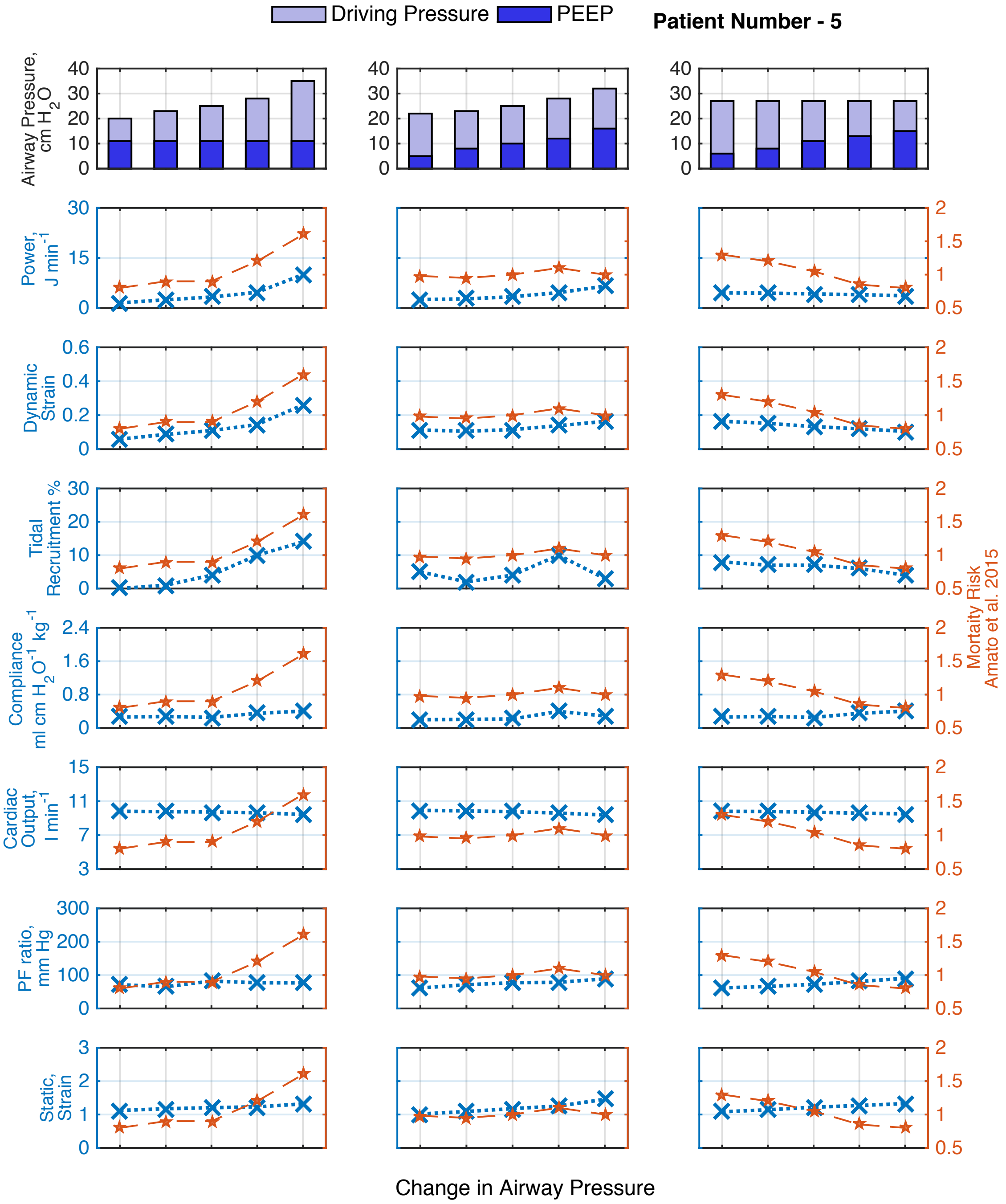

Driving Pressure
PEEP
Patient Number - 5
Airway Pressure, cm H2O
Power, J min-1
Dynamic Strain
Tidal Recruitment %
Compliance ml cm H2O-1 kg-1
Cardiac Output, l min-1
PF ratio, mm Hg
Static, Strain
Mortaity Risk Amato et al. 2015
Change in Airway Pressure

Patient 6.

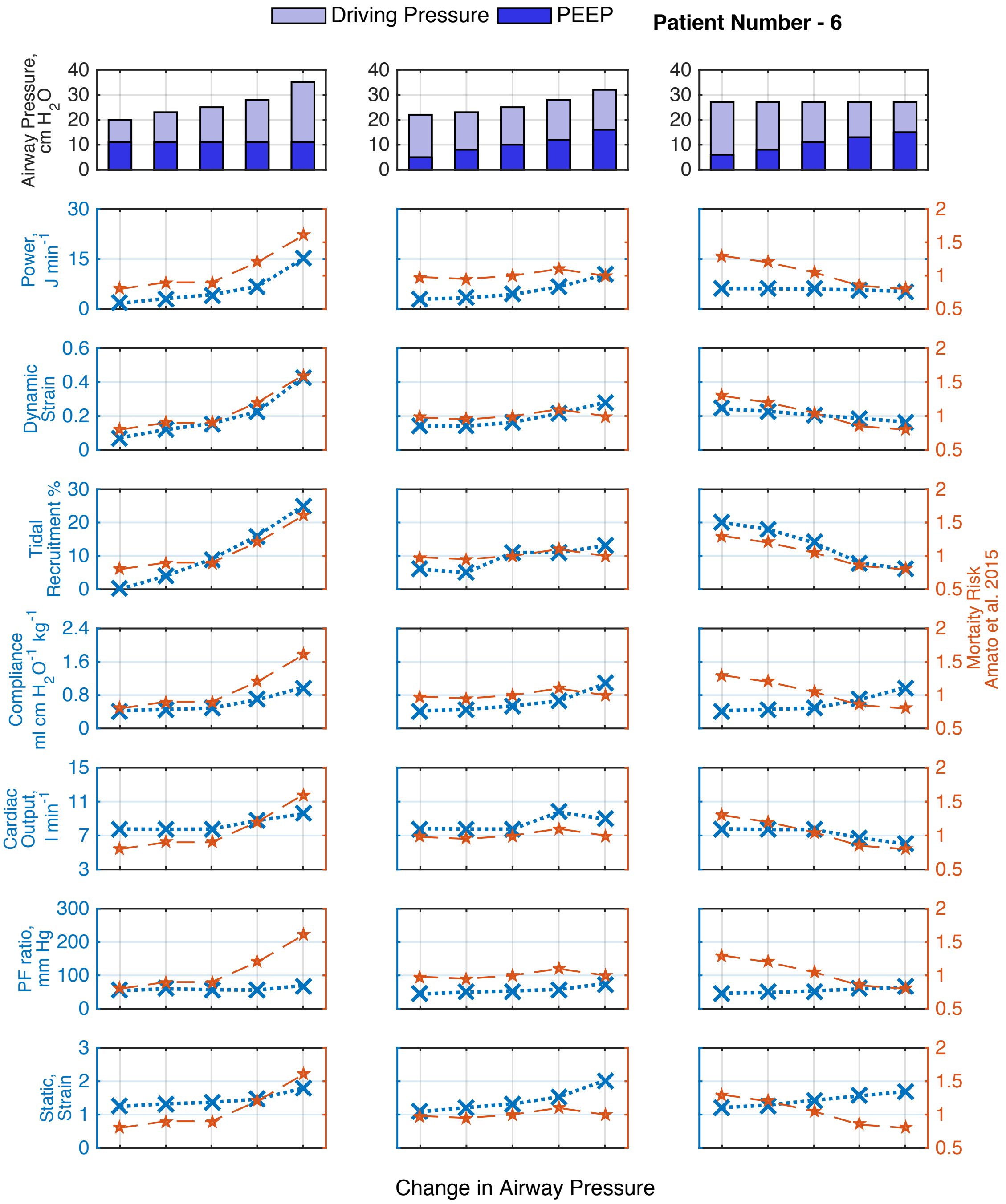
Driving Pressure
PEEP
Patient Number - 6
Airway Pressure, cm H_2O
Power, J min^{-1}
Dynamic Strain
Tidal Recruitment %
Compliance ml cm H_2O^{-1} kg^{-1}
Cardiac Output, l min^{-1}
PF ratio, mm Hg
Static, Strain
Mortaity Risk Amato et al. 2015
Change in Airway Pressure

Patient 7.

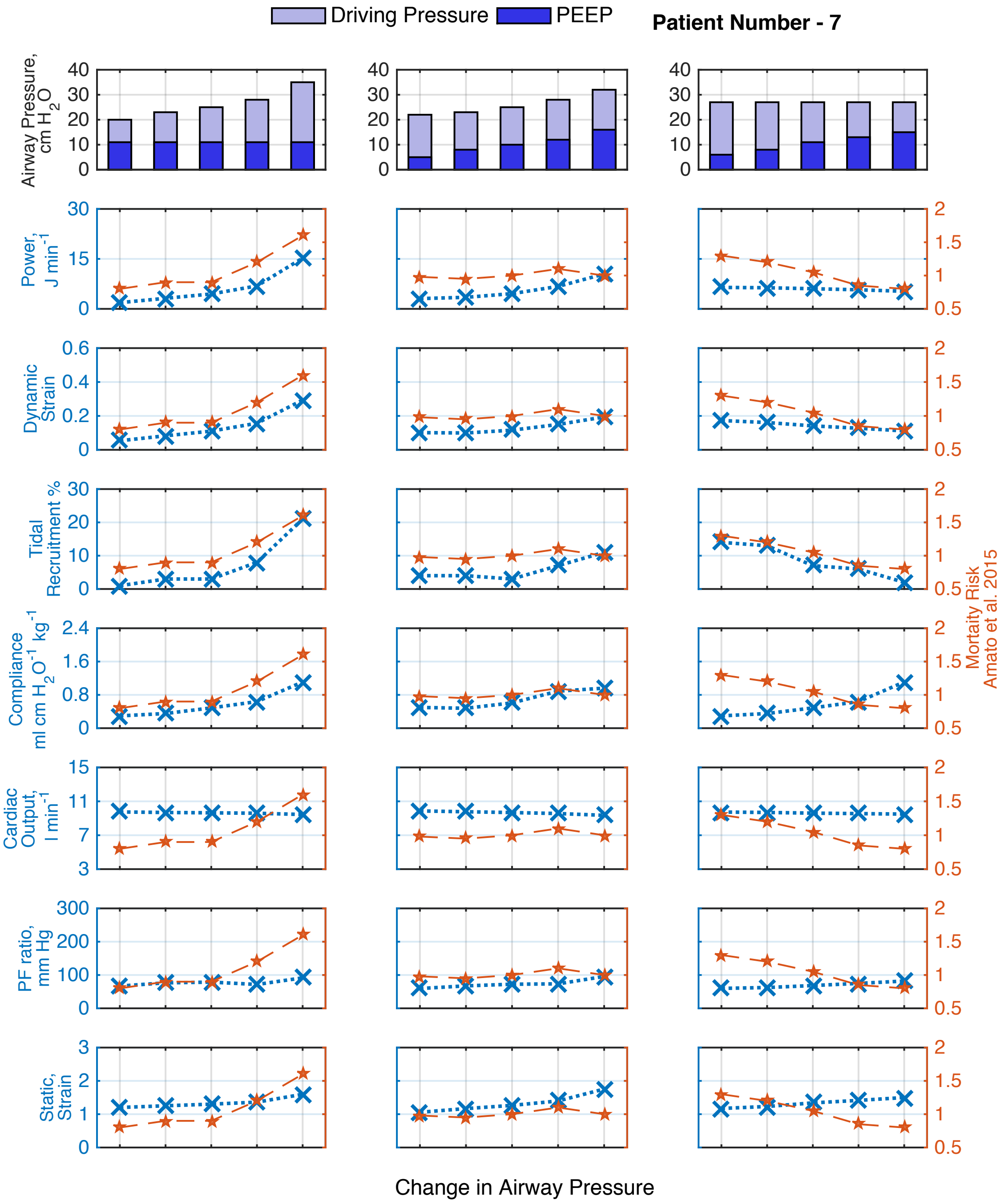
Driving Pressure
PEEP
Patient Number - 7
Airway Pressure, cm H2O
Power, J min-1
Dynamic Strain
Tidal Recruitment %
Compliance ml cm H2O-1 kg-1
Cardiac Output, l min-1
PF ratio, mm Hg
Static, Strain
Mortaity Risk
Amato et al. 2015
Change in Airway Pressure

Patient 8.

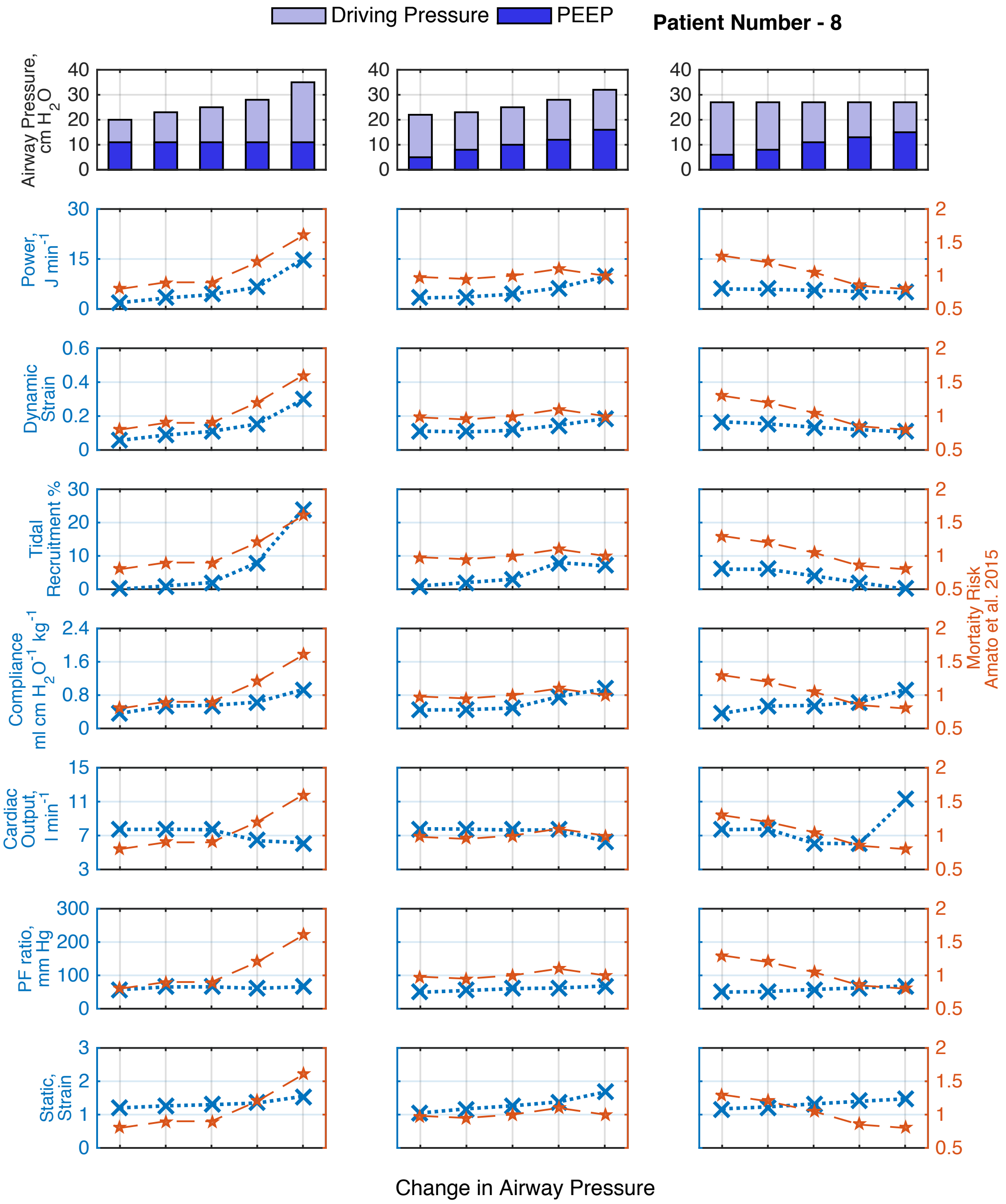

Driving Pressure
PEEP
Patient Number - 8
Airway Pressure, cm H_2O
Power, J min^{-1}
Dynamic Strain
Tidal Recruitment %
Compliance ml cm H_2O^{-1} kg^{-1}
Cardiac Output, l min^{-1}
PF ratio, mm Hg
Static, Strain
Mortaity Risk Amato et al. 2015
Change in Airway Pressure

Patient 9.

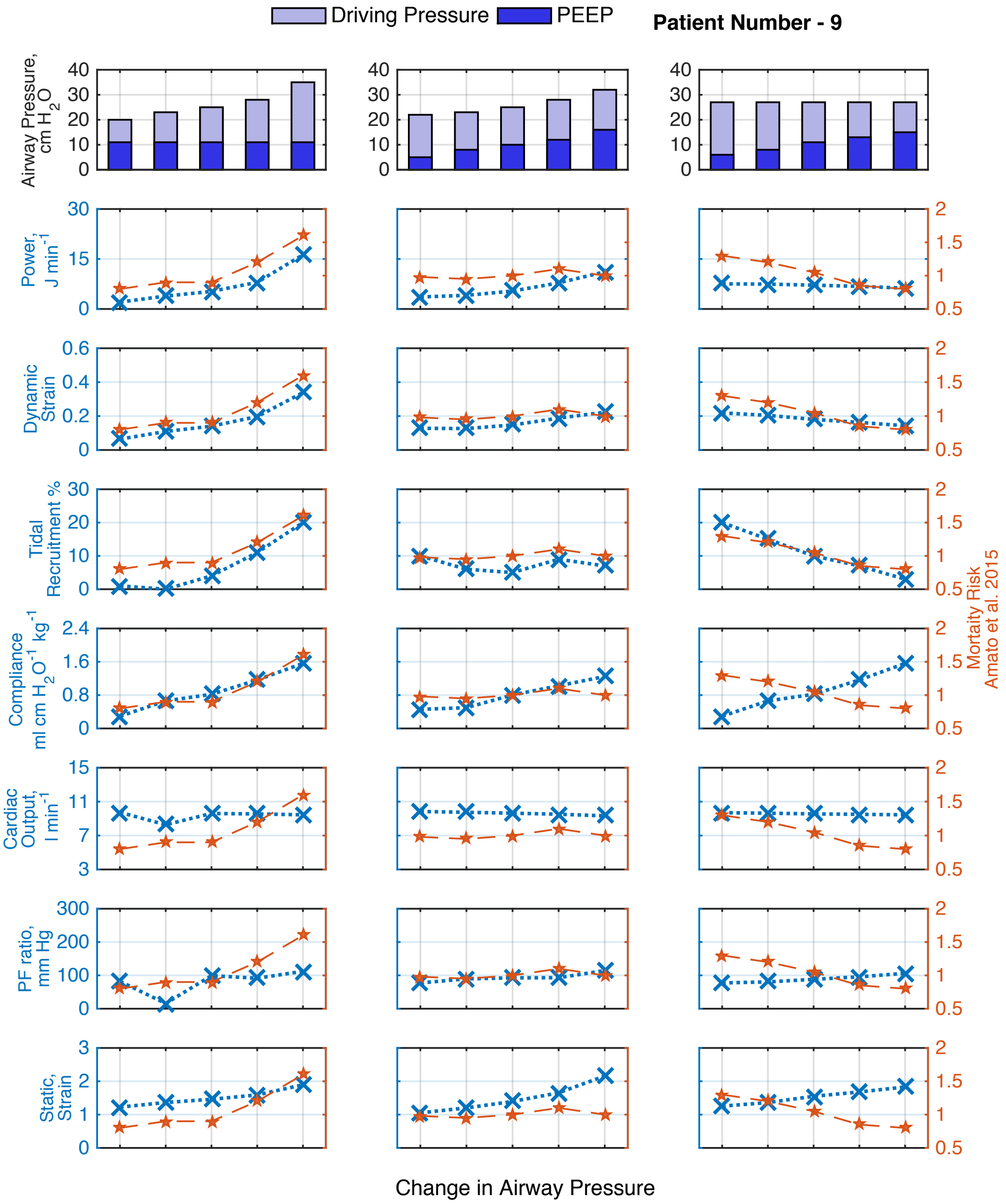
Driving Pressure
PEEP
Patient Number - 9
Airway Pressure, cm H_2O
Power, J min^{-1}
Dynamic Strain
Tidal Recruitment %
Compliance ml cm H_2O^{-1} kg^{-1}
Cardiac Output, l min^{-1}
PF ratio, mm Hg
Static, Strain
Mortaity Risk Amato et al. 2015
Change in Airway Pressure

Patient 10.

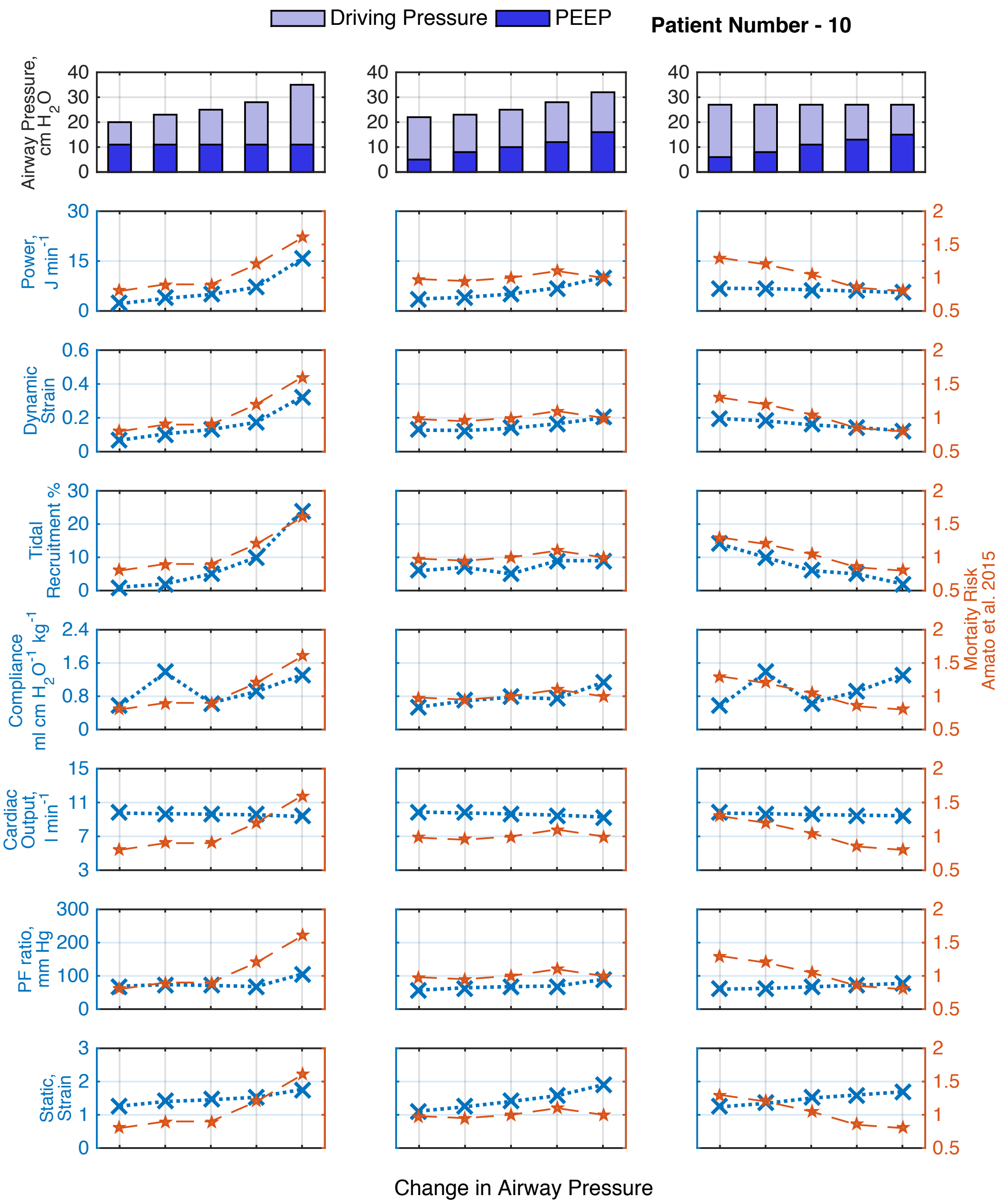

Driving Pressure
PEEP
Patient Number - 10
Airway Pressure, cm H_2O
Power, J min^{-1}
Dynamic Strain
Tidal Recruitment %
Compliance ml cm H_2O^{-1} kg^{-1}
Cardiac Output, l min^{-1}
PF ratio, mm Hg
Static, Strain
Mortaity Risk Amato et al. 2015
Change in Airway Pressure

Patient 11.

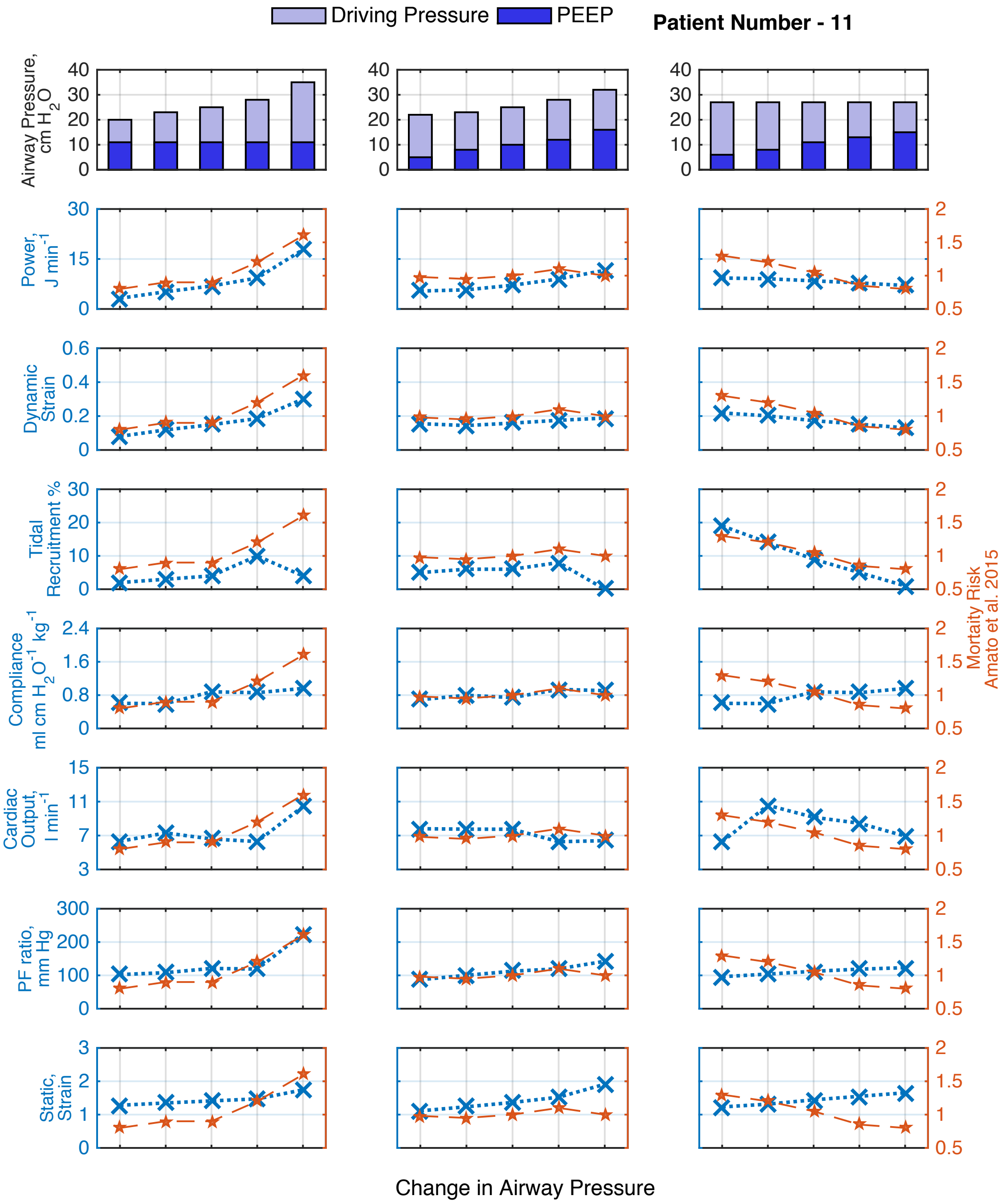

Driving Pressure
PEEP
Patient Number - 11
Airway Pressure, cm H_2O
Power, J min^{-1}
Dynamic Strain
Tidal Recruitment %
Compliance ml cm H_2O^{-1} kg^{-1}
Cardiac Output, l min^{-1}
PF ratio, mm Hg
Static, Strain
Mortaity Risk Amato et al. 2015
Change in Airway Pressure

Patient 12.

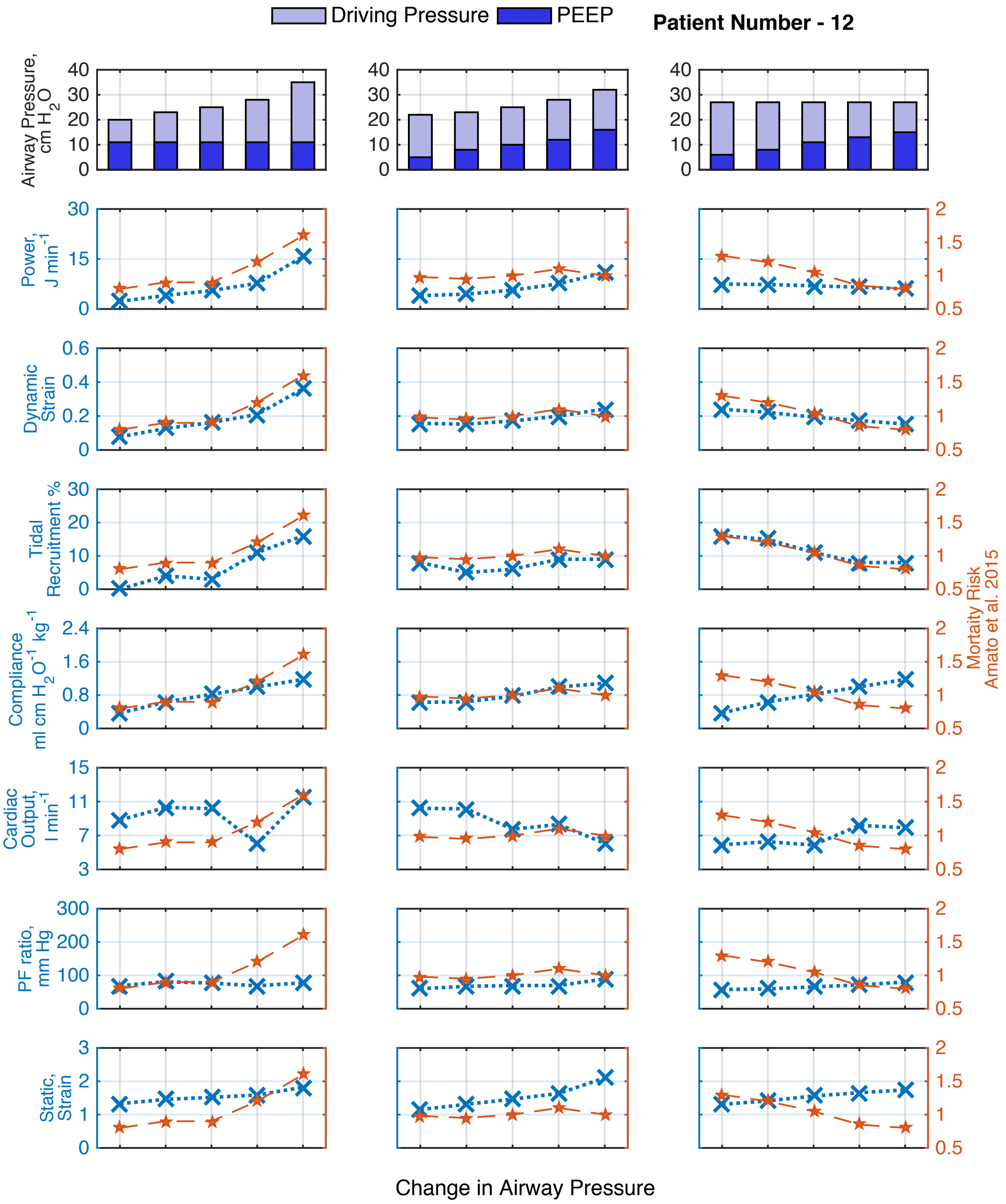

Driving Pressure
PEEP
Patient Number - 12
Airway Pressure, cm H2O
Power, J min-1
Dynamic Strain
Tidal Recruitment %
Compliance ml cm H2O-1 kg-1
Cardiac Output, l min-1
PF ratio, mm Hg
Static, Strain
Mortaity Risk Amato et al. 2015
Change in Airway Pressure

Patient 13.

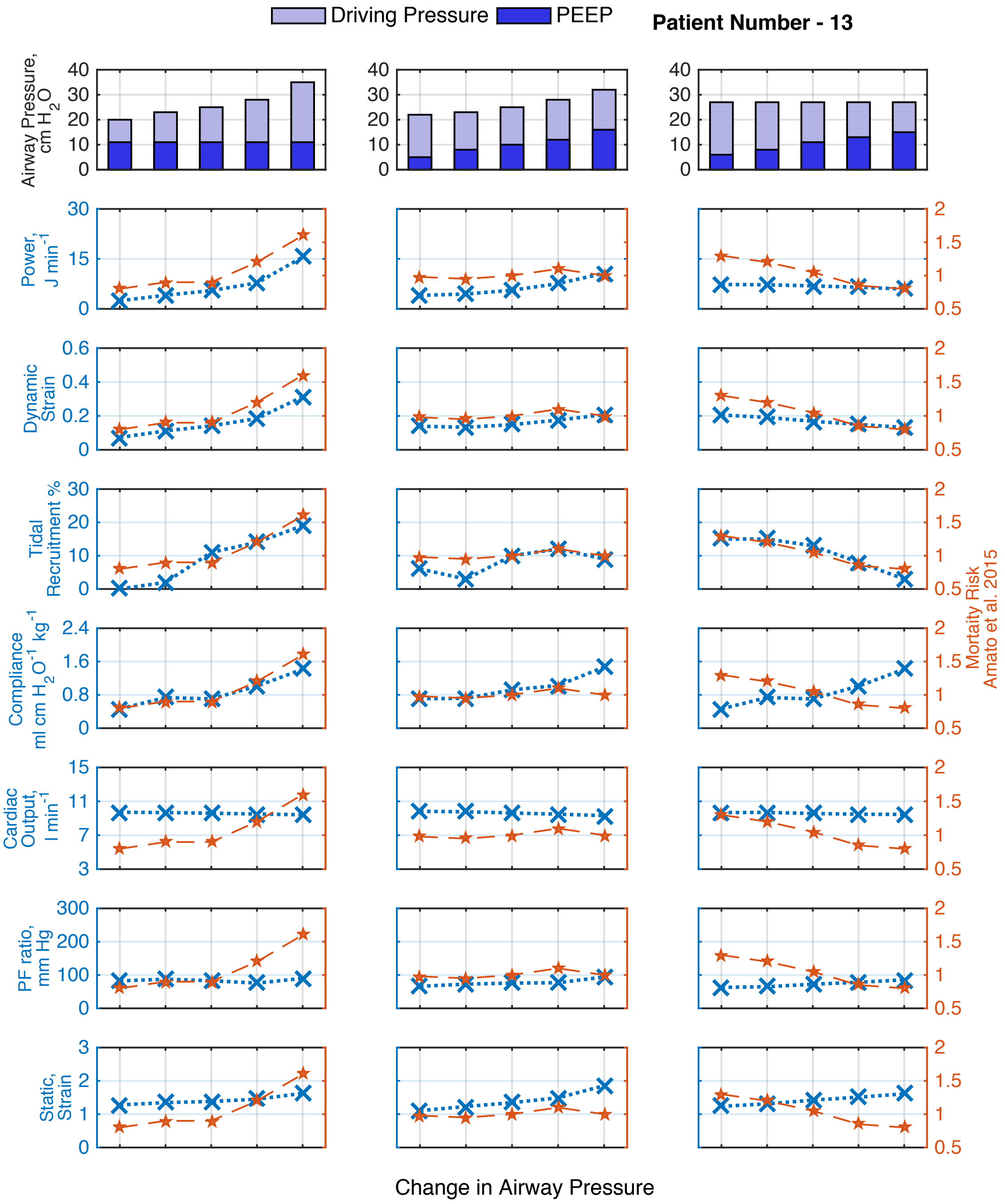
Driving Pressure
PEEP
Patient Number - 13
Airway Pressure, cm H2O
Power, J min-1
Dynamic Strain
Tidal Recruitment %
Compliance ml cm H2O-1 kg-1
Cardiac Output, l min-1
PF ratio, mm Hg
Static, Strain
Mortality Risk Amato et al. 2015
Change in Airway Pressure

Patient 14.

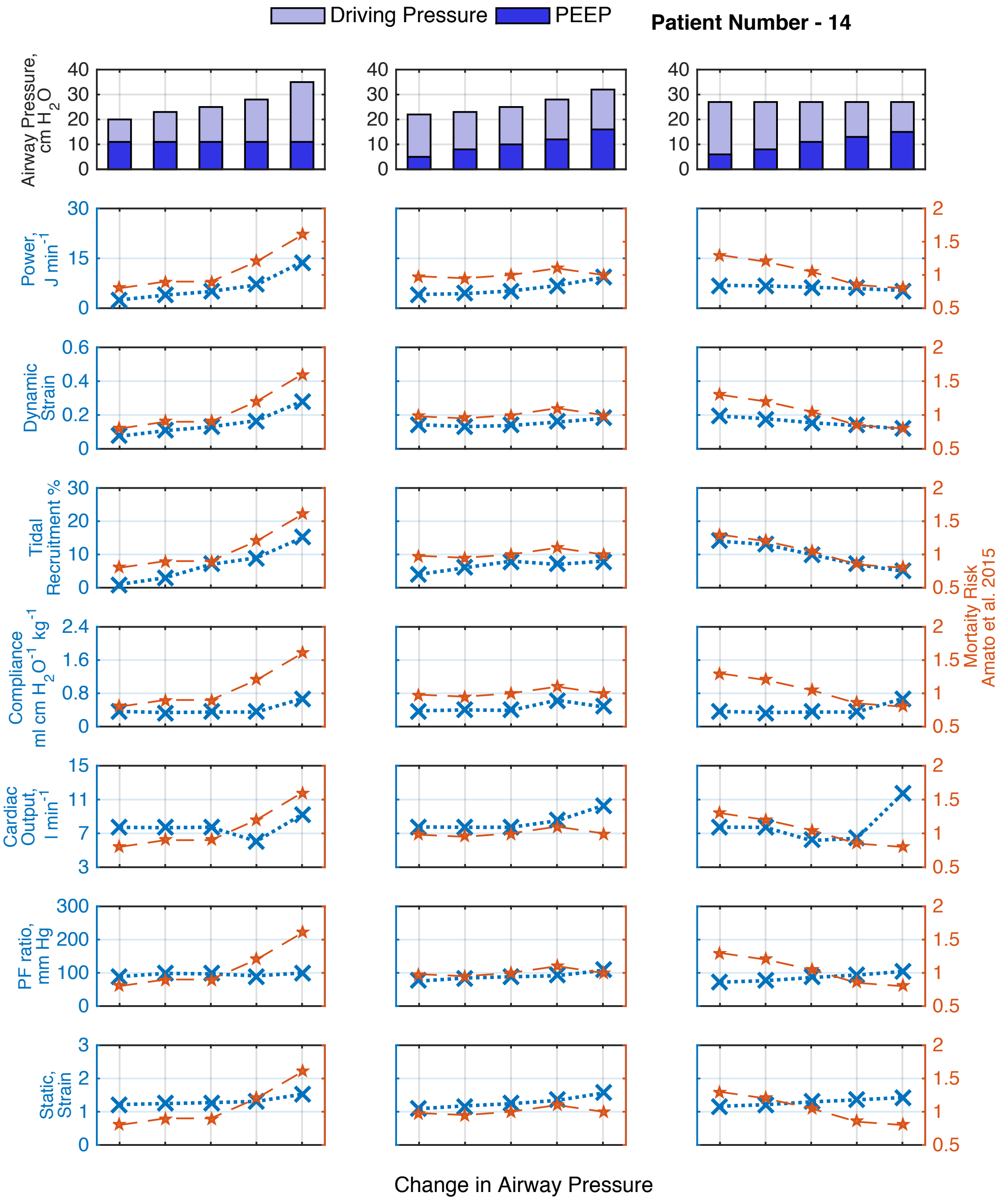

Driving Pressure
PEEP
Patient Number - 14
Airway Pressure, cm H_2O
Power, J min^{-1}
Dynamic Strain
Tidal Recruitment %
Compliance ml cm H_2O^{-1} kg^{-1}
Cardiac Output, l min^{-1}
PF ratio, mm Hg
Static, Strain
Mortaity Risk
Amato et al. 2015
Change in Airway Pressure

Patient 15.

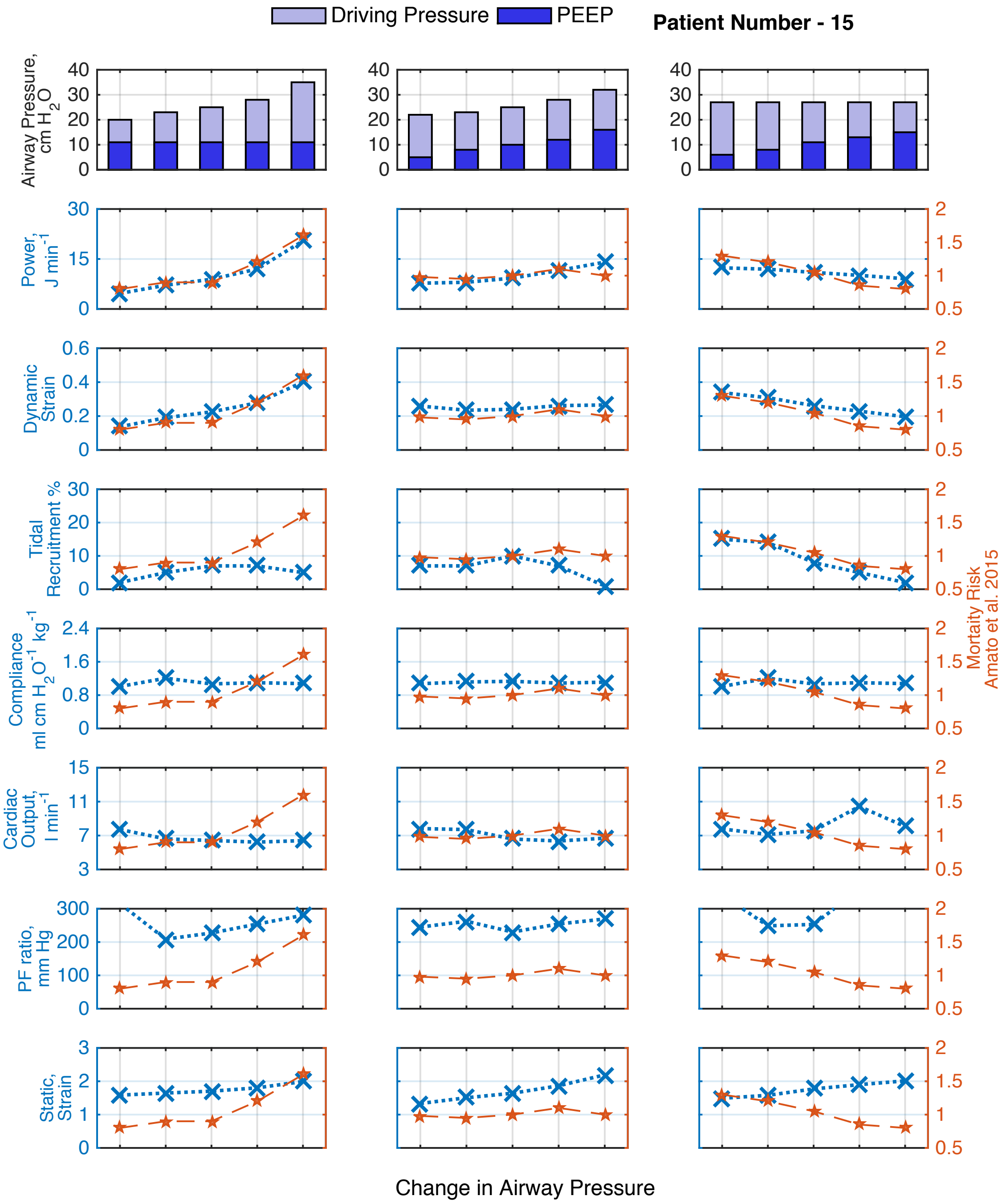
Driving Pressure
PEEP
Patient Number - 15
Airway Pressure, cm H2O
Power, J min-1
Dynamic Strain
Tidal Recruitment %
Compliance ml cm H2O-1 kg-1
Cardiac Output, l min-1
PF ratio, mm Hg
Static, Strain
Mortaity Risk Amato et al. 2015
Change in Airway Pressure

Patient 16.

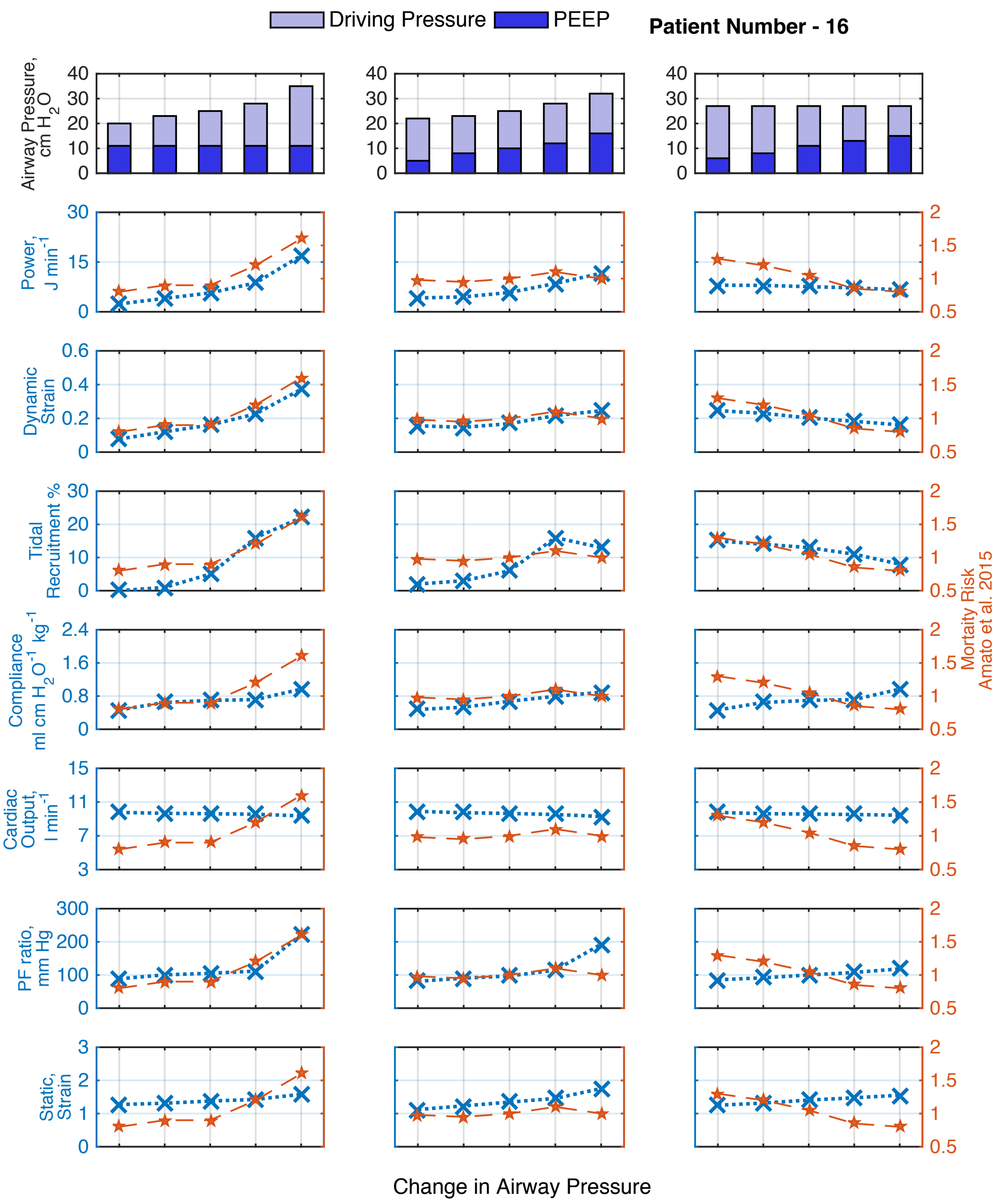
Driving Pressure
PEEP
Patient Number - 16
Airway Pressure, cm H_2O
Power, J min^{-1}
Dynamic Strain
Tidal Recruitment %
Compliance ml cm H_2O^{-1} kg^{-1}
Cardiac Output, l min^{-1}
PF ratio, mm Hg
Static, Strain
Mortaity Risk Amato et al. 2015
Change in Airway Pressure

Patient 17.

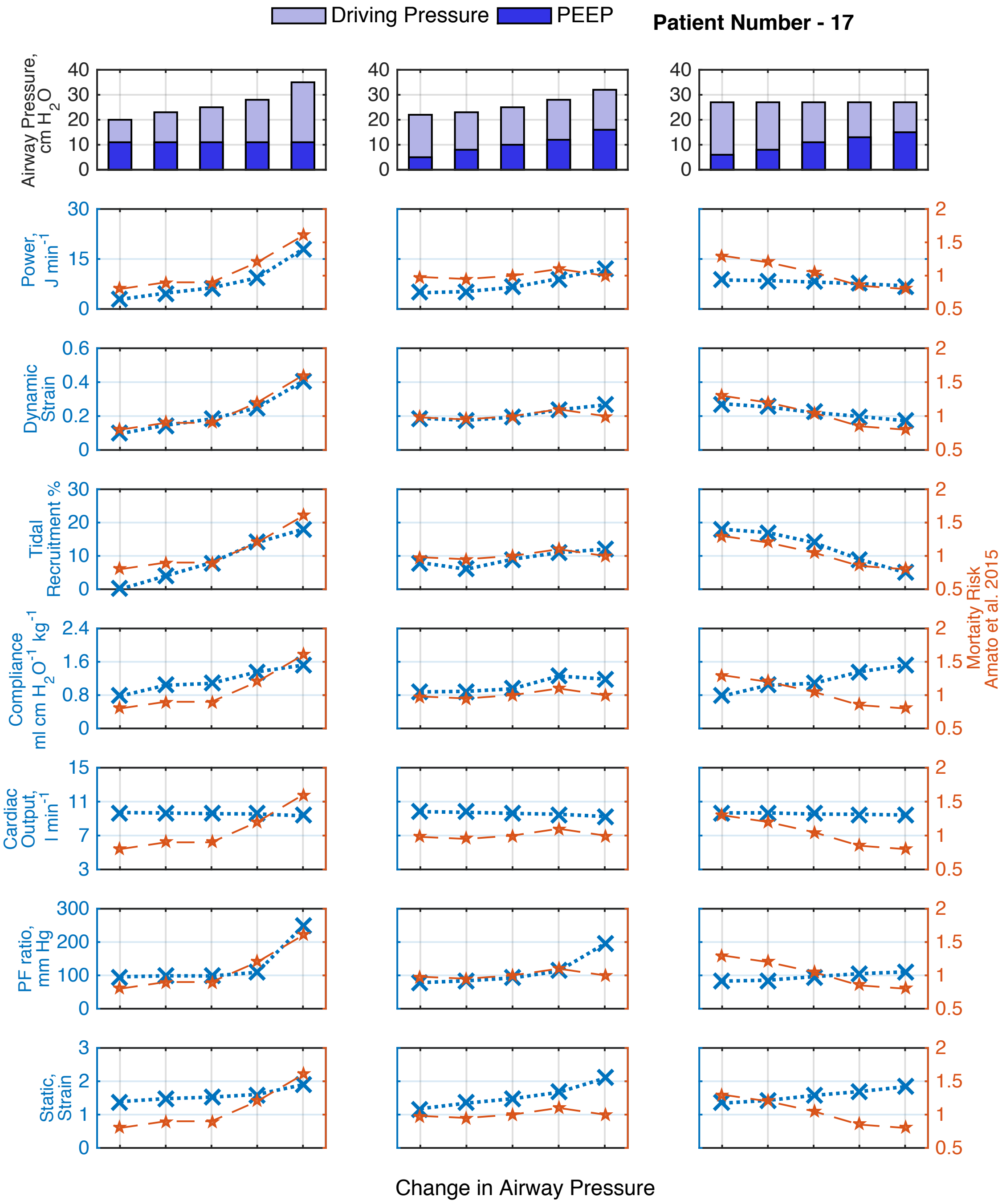
Driving Pressure
PEEP
Patient Number - 17
Airway Pressure, cm H_2O
Power, J min^{-1}
Dynamic Strain
Tidal Recruitment %
Compliance ml cm H_2O^{-1} kg^{-1}
Cardiac Output, l min^{-1}
PF ratio, mm Hg
Static, Strain
Mortality Risk
Amato et al. 2015
Change in Airway Pressure

Patient 18.

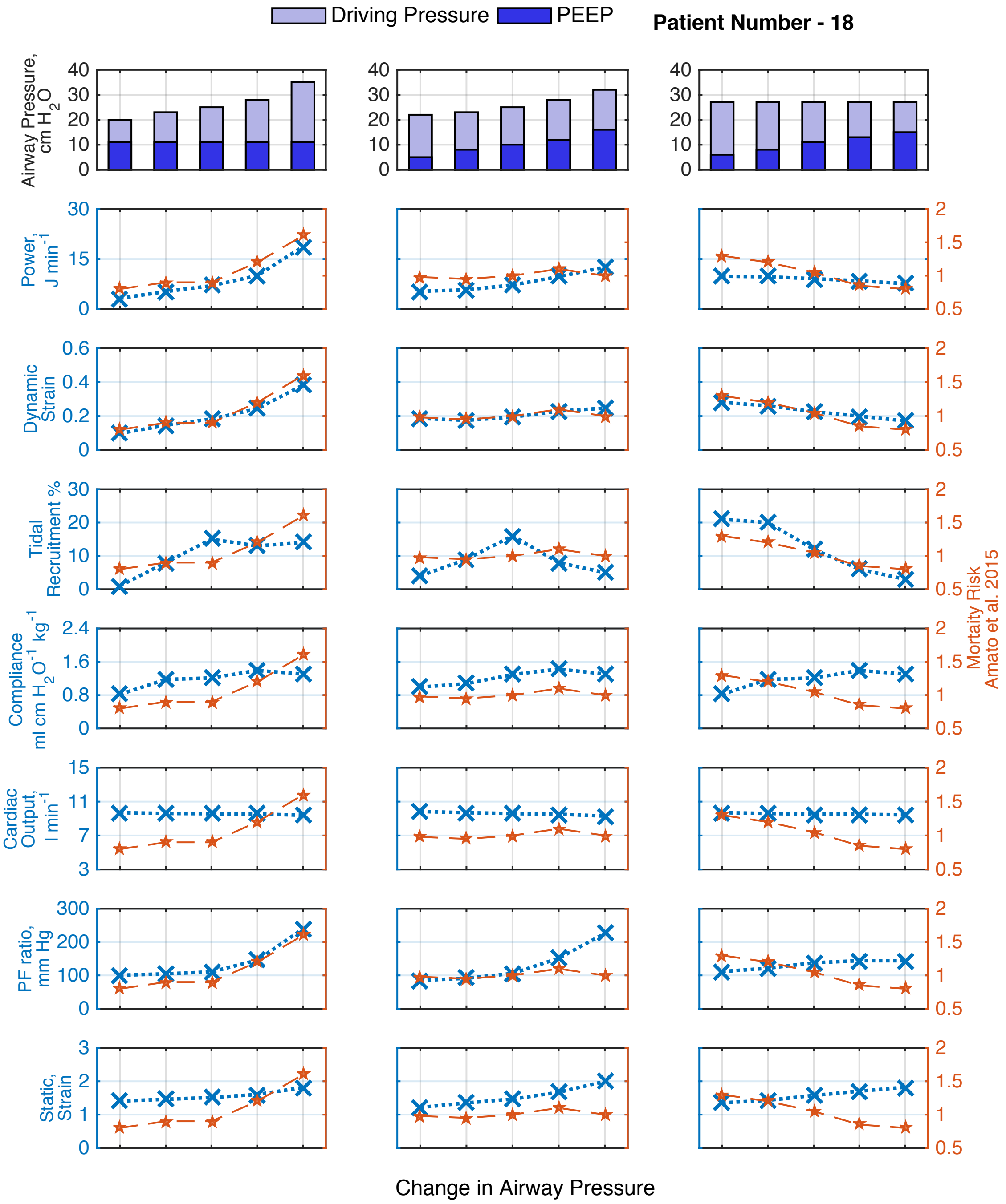

Driving Pressure
PEEP
Patient Number - 18
Airway Pressure, cm H_2O
Power, J min^{-1}
Dynamic Strain
Tidal Recruitment %
Compliance ml cm H_2O^{-1} kg^{-1}
Cardiac Output, l min^{-1}
PF ratio, mm Hg
Static, Strain
Mortality Risk Amato et al. 2015
Change in Airway Pressure

Patient 19.

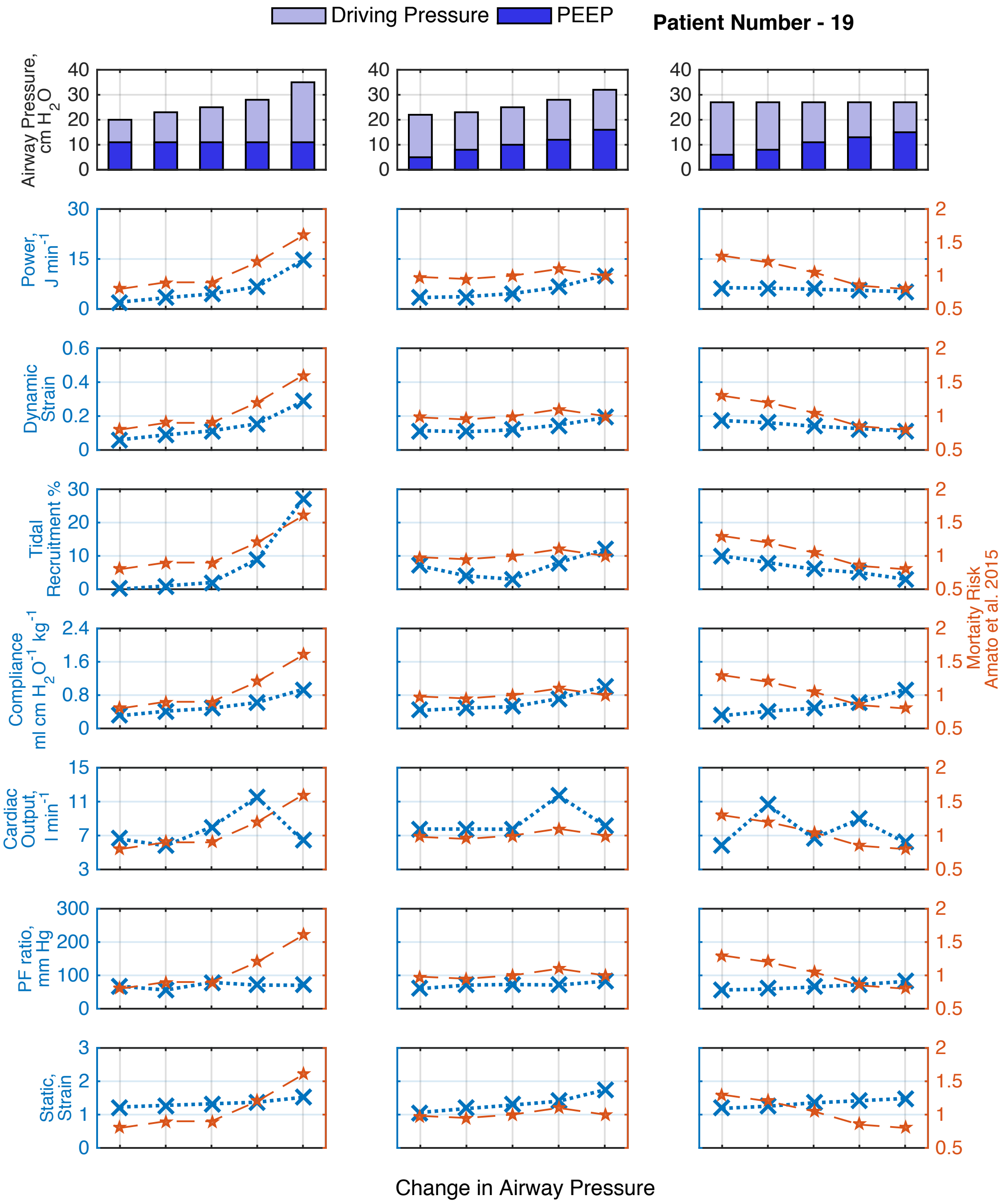
Driving Pressure
PEEP
Patient Number - 19
Airway Pressure, cm H_2O
Power, J min-1
Dynamic Strain
Tidal Recruitment %
Compliance ml cm H_2O^{-1} kg-1
Cardiac Output, l min-1
PF ratio, mm Hg
Static, Strain
Mortality Risk Amato et al. 2015
Change in Airway Pressure

Patient 20.

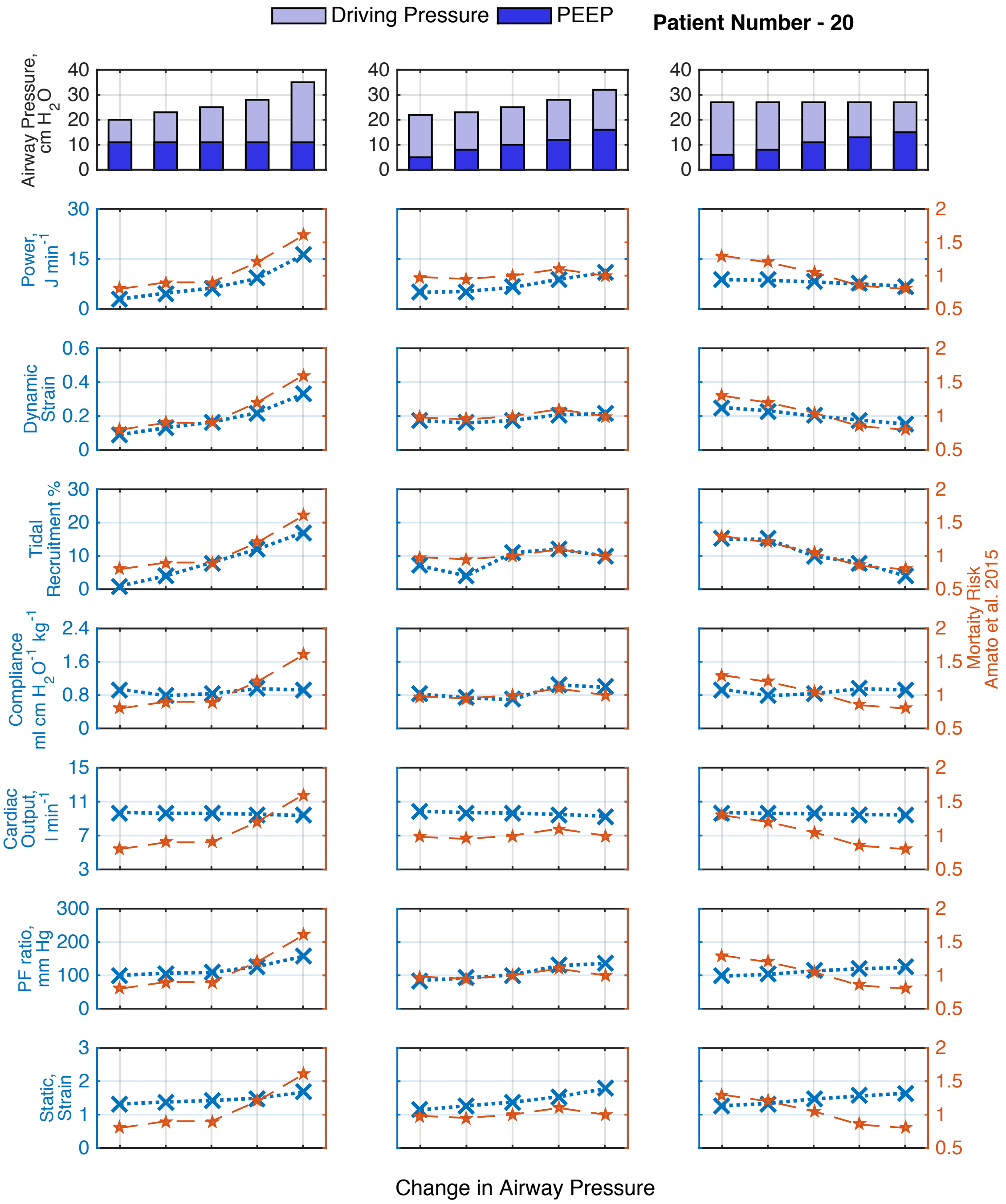
Driving Pressure
PEEP
Patient Number - 20
Airway Pressure, cm H_2O
Power, J min^{-1}
Dynamic Strain
Tidal Recruitment %
Compliance ml cm H_2O^{-1} kg^{-1}
Cardiac Output, l min^{-1}
PF ratio, mm Hg
Static, Strain
Mortaity Risk
Amato et al. 2015
Change in Airway Pressure

Patient 21.

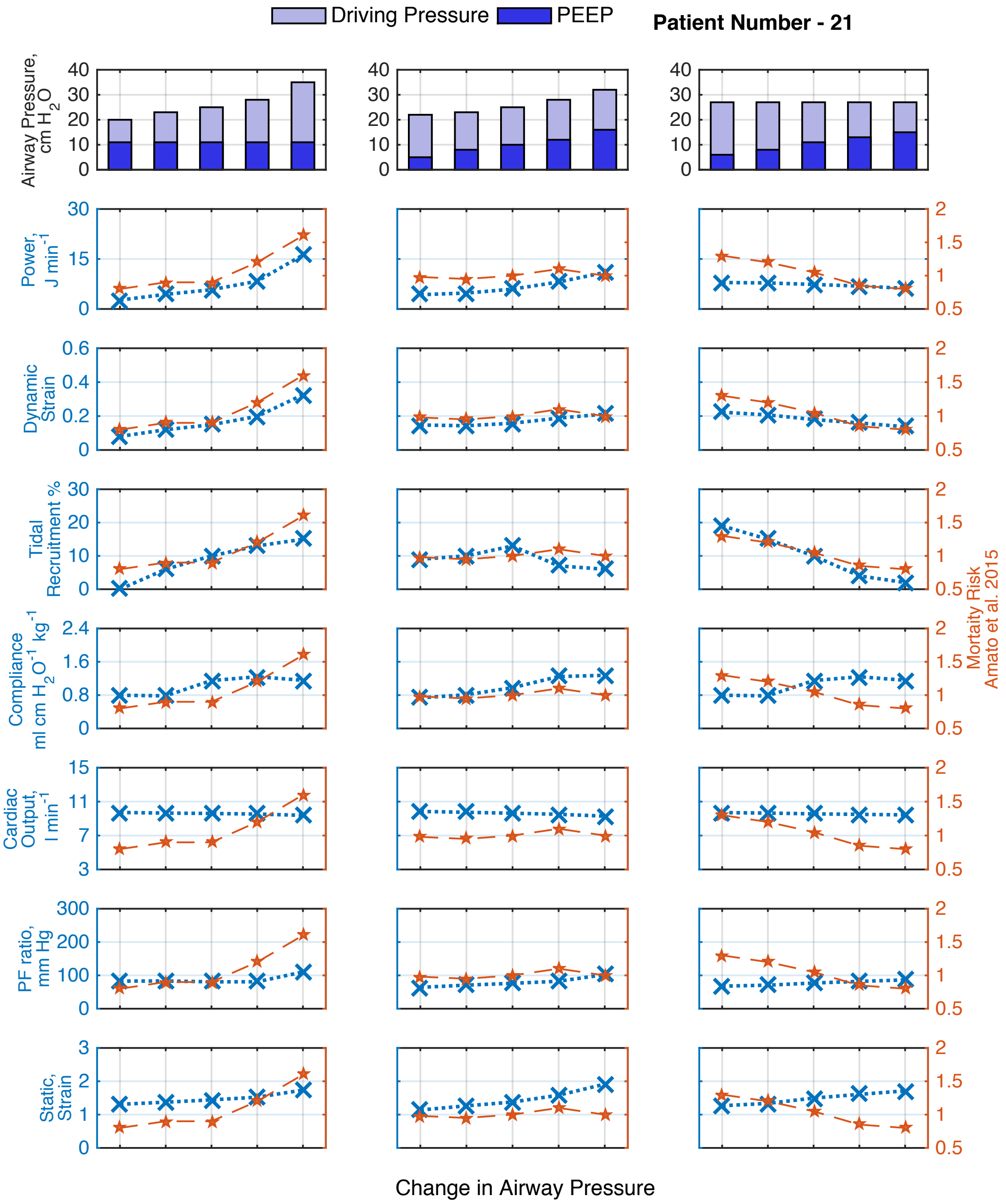
Driving Pressure
PEEP
Patient Number - 21
Airway Pressure, cm H2O
Power, J min-1
Dynamic Strain
Tidal Recruitment %
Compliance ml cm H2O-1 kg-1
Cardiac Output, l min-1
PF ratio, mm Hg
Static, Strain
Mortality Risk Amato et al. 2015
Change in Airway Pressure

Patient 22.

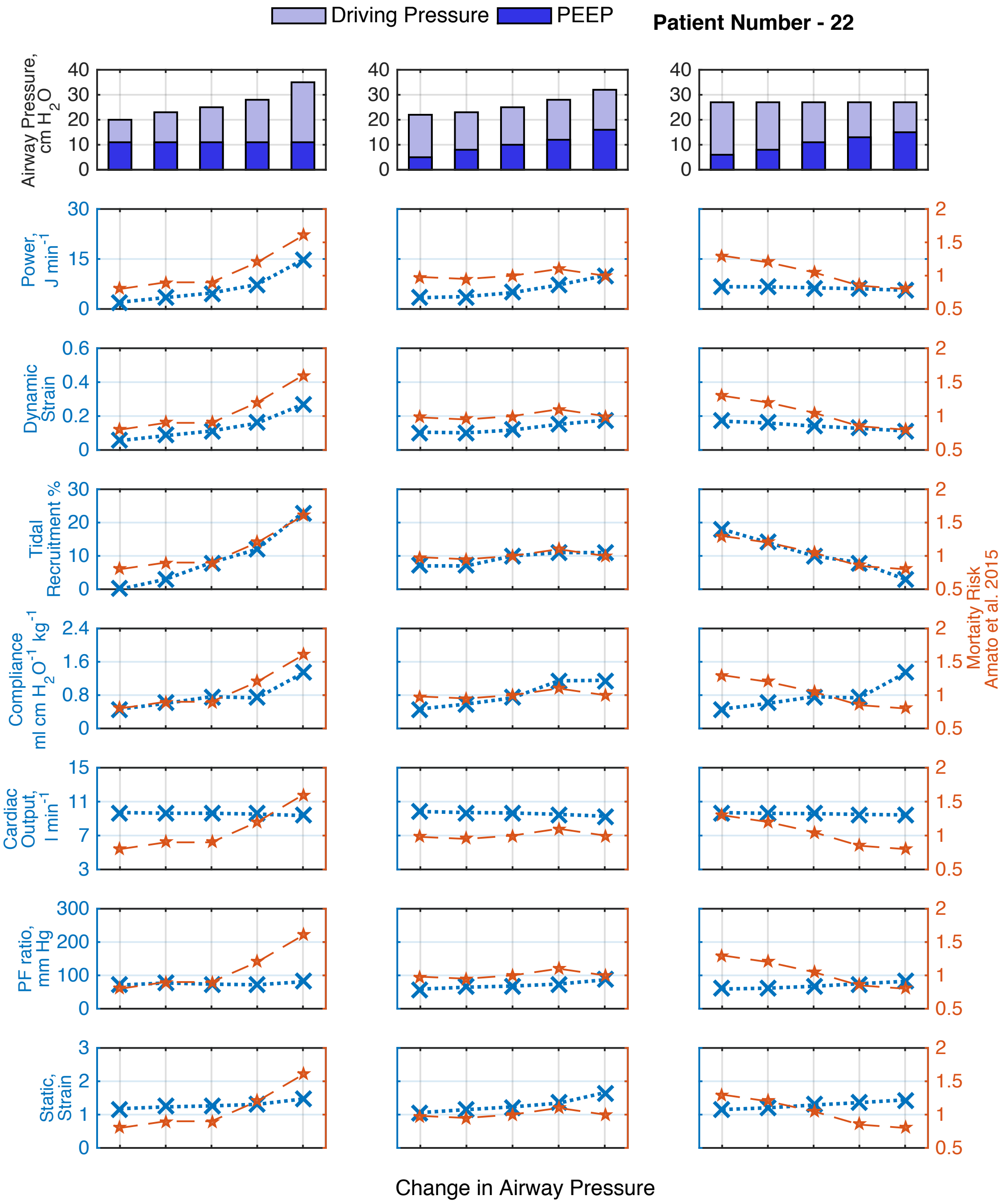
Driving Pressure
PEEP
Patient Number - 22
Airway Pressure, cm H_2O
Power, J min^{-1}
Dynamic Strain
Tidal Recruitment %
Compliance ml cm H_2O^{-1} kg^{-1}
Cardiac Output, l min^{-1}
PF ratio, mm Hg
Static, Strain
Mortality Risk Amato et al. 2015
Change in Airway Pressure

Patient 23.

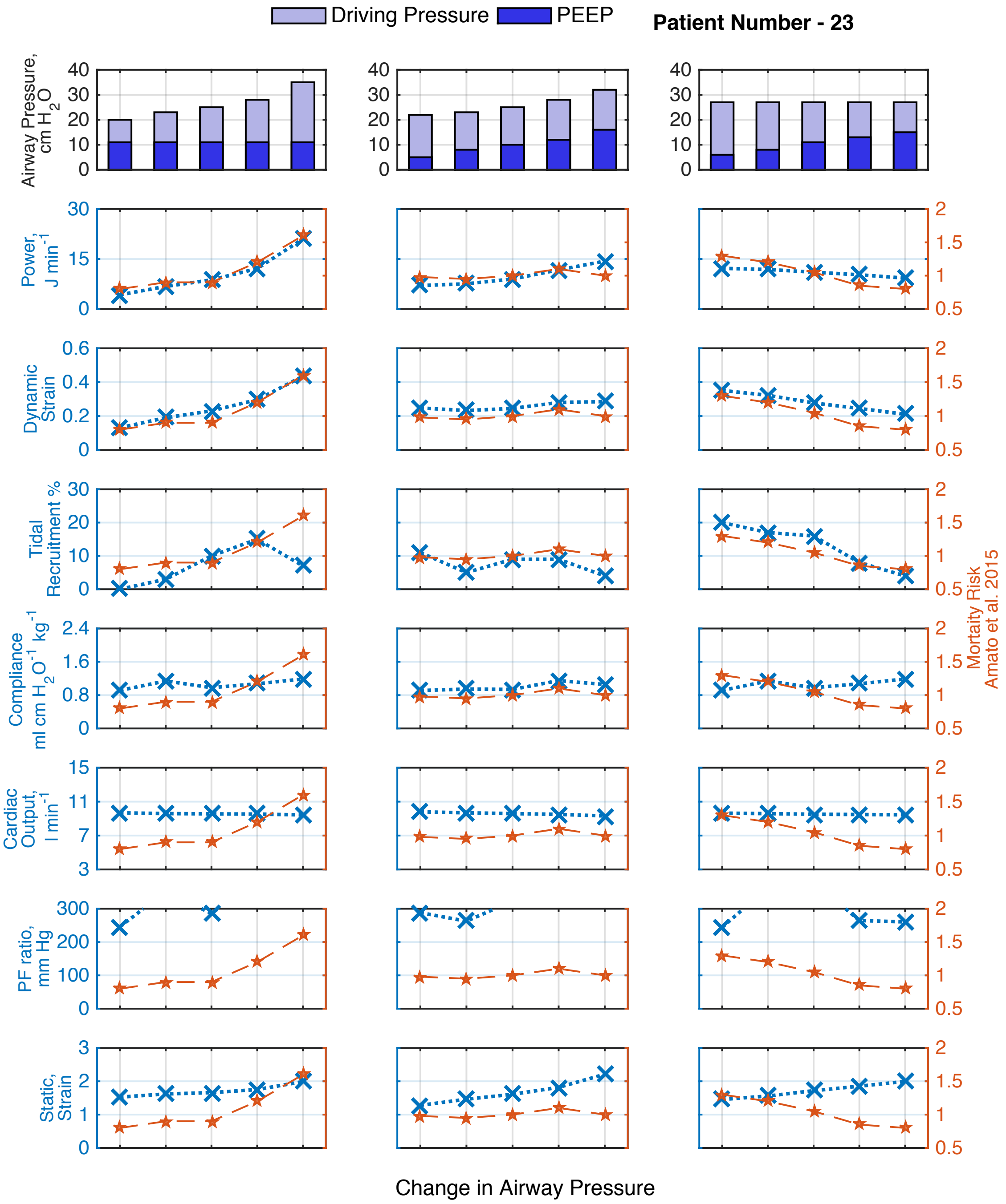
Driving Pressure
PEEP
Patient Number - 23
Airway Pressure, cm H2O
Power, J min-1
Dynamic Strain
Tidal Recruitment %
Compliance ml cm H2O-1 kg-1
Cardiac Output, l min-1
PF ratio, mm Hg
Static, Strain
Mortality Risk Amato et al. 2015
Change in Airway Pressure

Patient 24.

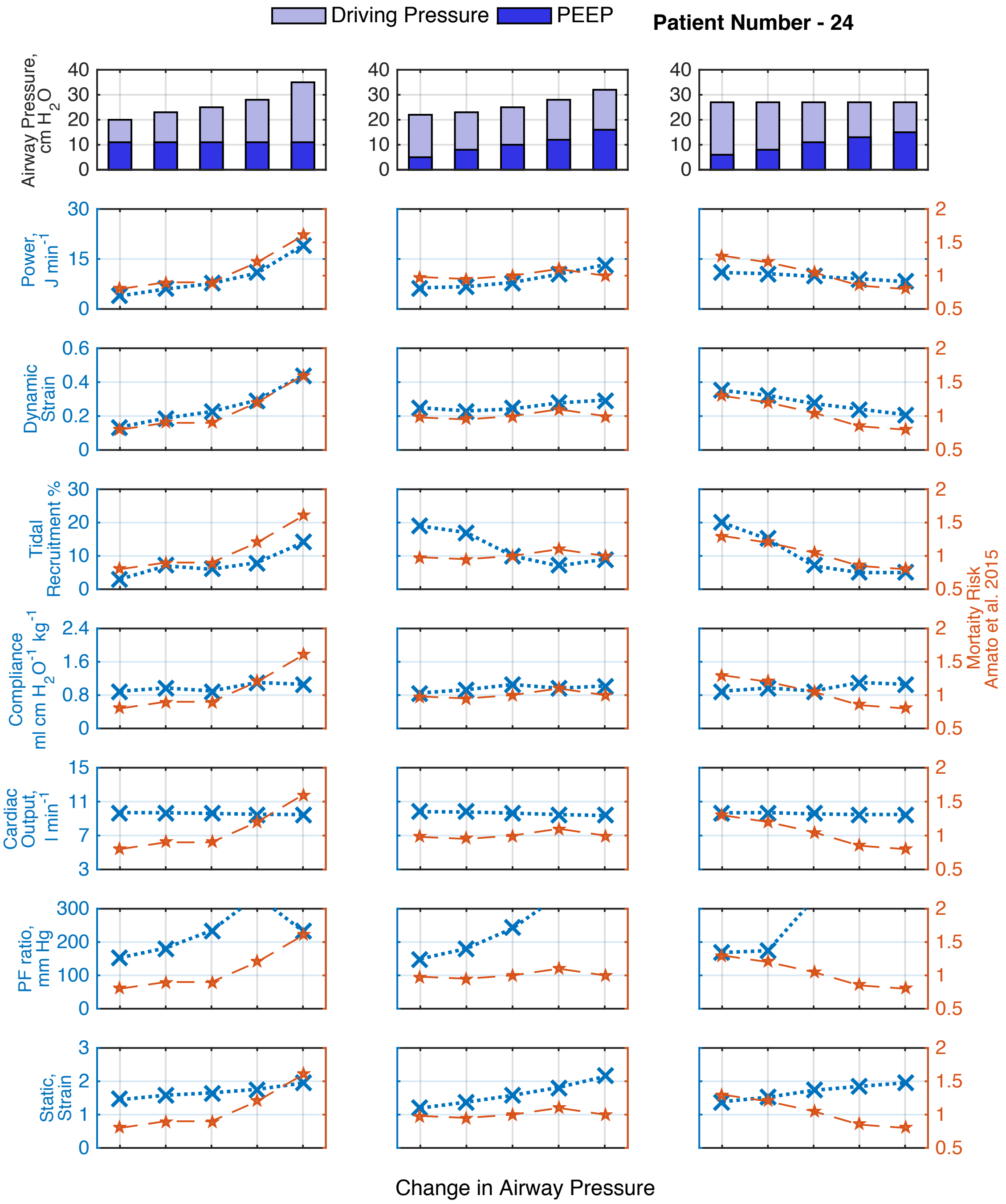
Driving Pressure
PEEP
Patient Number - 24
Airway Pressure, cm H_2O
Power, J min^{-1}
Dynamic Strain
Tidal Recruitment %
Compliance ml cm H_2O^{-1} kg^{-1}
Cardiac Output, l min^{-1}
PF ratio, mm Hg
Static, Strain
Mortality Risk Amato et al. 2015
Change in Airway Pressure

Patient 25.

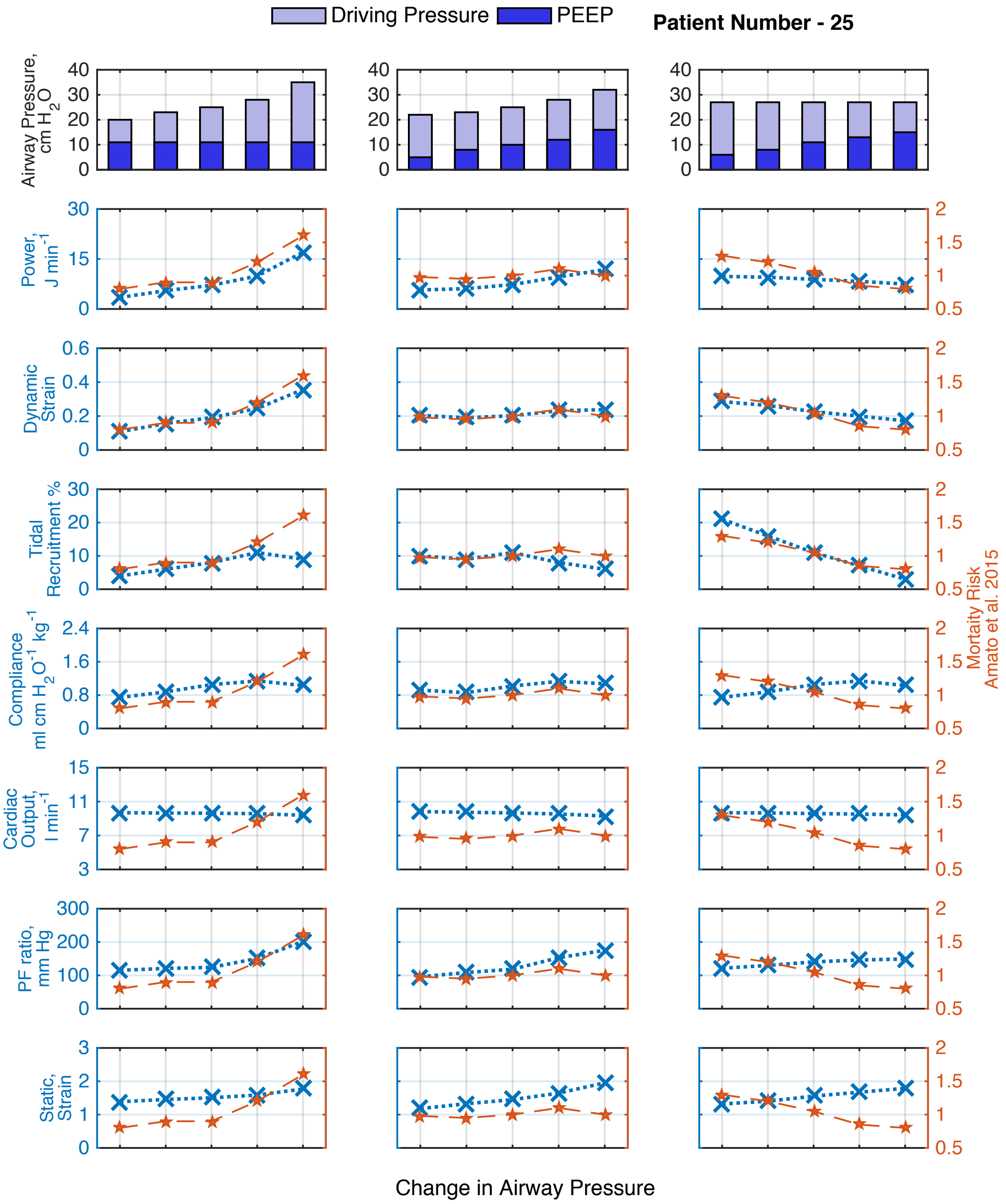
Driving Pressure
PEEP
Patient Number - 25
Airway Pressure, cm H_2O
Power, J min^{-1}
Dynamic Strain
Tidal Recruitment %
Compliance ml cm H_2O^{-1} kg^{-1}
Cardiac Output, l min^{-1}
PF ratio, mm Hg
Static, Strain
Mortality Risk Amato et al. 2015
Change in Airway Pressure
